# Supplementary material for: Non-aromatic annulene-based aggregation-induced emission system via aromaticity reversal process
Source: Nat Commun. 2019 Jul 4;10:2952. doi: 10.1038/s41467-019-10818-5 (PMC6609648; doi:10.1038/s41467-019-10818-5)
Supplement: Supplementary file 1 — Supplementary Information [file 41467_2019_10818_MOESM1_ESM.pdf]

Supplementary Information

Non-aromatic annulene-based aggregation-induced emission system via aromaticity reversal process

Zhao *et al*.

Methods

Femtosecond transient absorption (fs-TA) experiment. Fs-TA measurement was done by using a 1000 Hz femtosecond regenerative amplified Ti:sapphire laser system (Maitai) in which the amplifier was seeded with the 120 fs laser pulses from an oscillator laser system. The laser probe pulse was produced by utilizing ~5% of the amplified 800 nm laser pulses to generate a white-light continuum (430-750 nm) in a saphire crystal and then the probe beam was split into two parts before penetrating the sample. One probe laser beam went through the sample while the other probe laser beam went to the reference spectrometer in order to monitor the fluctuations in the probe beam intensity. For the present experiments, the compounds in THF solution were excited by a 400 nm pump beam (the second harmonic of the fundamental 800 nm from the regenerative amplifier). The 1 mL solutions were studied in a 2 mm path-length cuvette with an absorbance of 0.5 at 400 nm throughout the data acquisition.

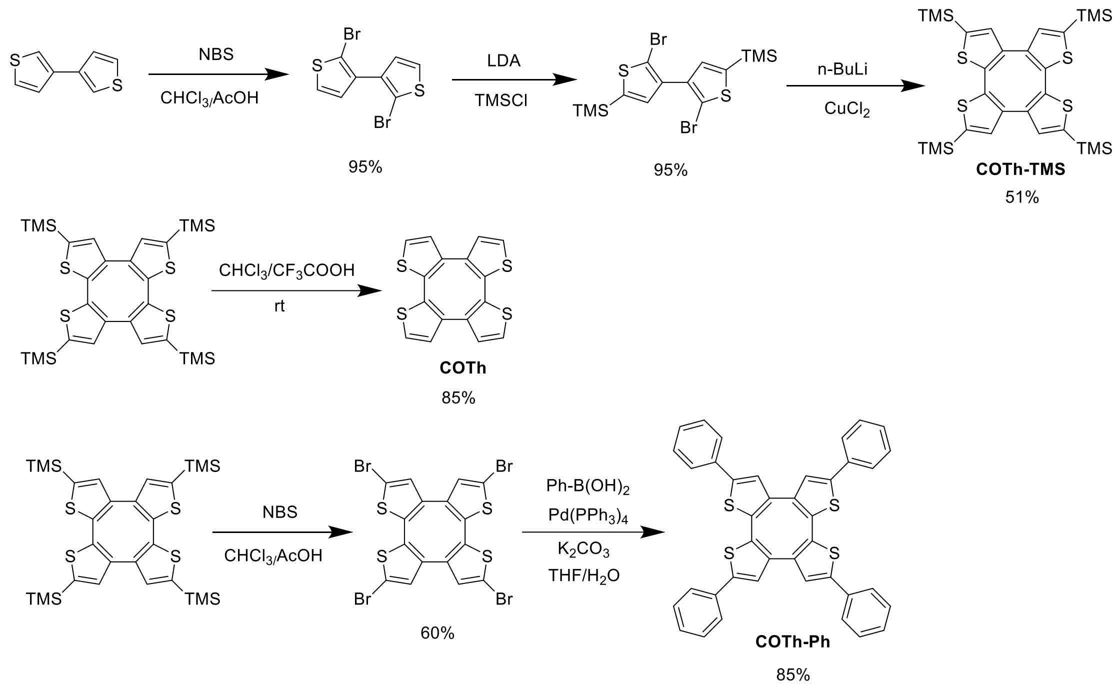

Supplementary Figure 1. Synthetic routes of COTh-TMS, COTh and COTh-Ph. Compounds COT-TMS, COT and COT-Ph were synthesized according to the literature method[1,2]

Synthesis of 3. A mixture of compound 4Br-COTh (50 mg, 0.078 mmol), $Pd(OAc)_2$ (0.17 mg, 1% mmol), 4-methoxyphenylboronic acid (152 mg, 1 mmol) and K_2CO_3 (107 mg, 0.78 mmol) were added successively into anhydrous N,N-dimethylformamide under N_2. The reaction mixture was stirred at 120°C for 8h under N_2. After cooling to room temperature, saturated NH_4Cl(aq) was added to the mixture and the precipitated product was filtered and collect. The crude product was purified by column chromatography (Hex/DCM = 1/1) and recrystallization in toluene to afford the pure **3** (41 mg) in a yield of 70%. ^{1}H NMR (400 MHz, $CDCl_3$, 298k), δ (ppm): 7.54–7.51 (d, J = 8 Hz, 8H), 7.13 (s, 4H), 6.93–6.91 (d, J = 8 Hz, 8H), 3.84 (s, 12H). ^{13}C NMR (150 MHz, $C_2D_2Cl_4$, 298k), δ (ppm): 160.9, 146.6, 138.8, 131.9, 128.3, 127.7, 126.3, 115.9, 56.9. HRMS (MALDI-TOF): m/z: [M]+ calcd for $C_{44}H_{32}O_4S_4$, 752.1183; found, 752.1129.

Synthesis of 2. To a solution of compound **3** (23 mg, 0.03 mmol) in dichloromethane (3 mL) at 0 °C, Br_3B (0.45 mL, 0.45 mmol, 1M in Hexane) was added under N_2 atmosphere, the color of the mixture become green immediately. Keeping stirring at 0

°C for 30 min and then increasing the temperature to room temperature overnight. Adding methanol to quench the reaction, and then adding water to precipitate the crude product, the precipitated product was filtered and collect. The crude product was purified by column chromatography (EA/DCM = 1/3) to afford the pure **3** (13 mg) in a yield of 62%. ^{1}H NMR (400 MHz, $CDCl_3$, 298k), *δ* (ppm): 8.72 (s, 1H), 7.58–7.56 (d, *J* = 8 Hz, 2H), 7.35 (s, 1H), 6.93–6.91 (d, *J* = 8 Hz, 2H). ^{13}C NMR (100 MHz, $CDCl_3$, 298k), *δ* (ppm): 157.8, 145.8, 137.8, 129.7, 127.0, 125.2, 124.8, 115.9. HRMS (MALDI-TOF): *m*/*z*: [M]+ calcd for $C_{40}H_{24}O_4S_4$, 696.0557; found, 696.0565.

Synthesis of 1. To a solution of 1,6-dibromohexane (31 mg, 0.126 mmol) and K_2CO_3 in acetone (3 mL), the solution of compound 2 (20 mg, 0.0287 mmol) was added dropwise within 2h, keeping reaction for overnight. Removing the solvent under vacuumed pressure, the crude product was purified by column chromatography with hexane as eluent, affording the pure **1** (20 mg) in a yield of 52%. ^{1}H NMR (400 MHz, $CDCl_3$, 298k), *δ* (ppm): 7.51–7.49 (d, *J* = 8 Hz, 2H), 7.11 (s, 1H), 6.90–6.88 (d, *J* = 8 Hz, 2H), 3.99–3.97 (d, *J* = 8 Hz, 1H), 3.96 (s, 1H), 3.44 (s, 1H), 3.43–3.41 (d, *J* = 8 Hz, 1H), 1.91–1.88 (m, 2H), 1.82–1.79 (m, 2H), 1.51 (m, 4H) . ^{13}C NMR (100 MHz, $CDCl_3$, 298k), *δ* (ppm): 158.4, 144.8, 136.9, 129.9, 126.3, 125.9, 124.1, 114.3, 67.2, 52.8, 33.2, 32.1, 28.5, 27.3, 24.7. HRMS (MALDI-TOF): *m*/*z*: [M]+ calcd for $C_{64}H_{68}Br_4O_4S_4$, 1344.0734; found, 1344.0747.

Synthesis of COTH-Py. To a solution of pyridine (3 mL), compound **1** (50 mg, 0.037 mmol) was added and reacted at room temperature for 12 h with a large amount of precipitate generating. The solid was filtered and collected, washed with DCM and acetone, affording the pure product (35 mg) in a yield of 57%. ^{1}H NMR (400 MHz, $CDCl_3$, 298k), *δ* (ppm): 9.01–8.09 (d, *J* = 8 Hz, 2H), 8.53 (m, 1H), 8.07–8.03 (t, *J* = 8 Hz, 2H), 7.40–7.37 (d, *J* = 12 Hz, 2H), 7.02 (s, 1H), 6.79–6.77(d, *J* = 8 Hz, 2H), 4.63–4.59 (t, *J* = 12 Hz, 2H) 3.80 (s, 2H), 1.97–1.92 (m, 2H), 1.65 (m, 2H), 1.38 (m, 4H) . ^{13}C NMR (100 MHz, $CDCl_3$, 298k), *δ* (ppm): 159.1, 145.4, 144.4, 137.5, 129.9, 126.6, 125.8, 124.7, 114.8, 67.6, 61.5, 31.0, 28.7, 25.5, 25.2. HRMS (ESI): *m*/*z*: $[M{-}4Br]^{+}$ calcd for $C_{84}H_{88}N_4O_4S_4$, 336.5; found, 336.6; $[M{-}3Br]^{+}$ calcd for

$C_{84}H_{88}N_4O_4S_4Br$, 475.2; found, 475.1; $[M{-}2Br]^+$ calcd for $C_{84}H_{88}N_4O_4S_4Br_2$, 752.8; found, 752.4; .

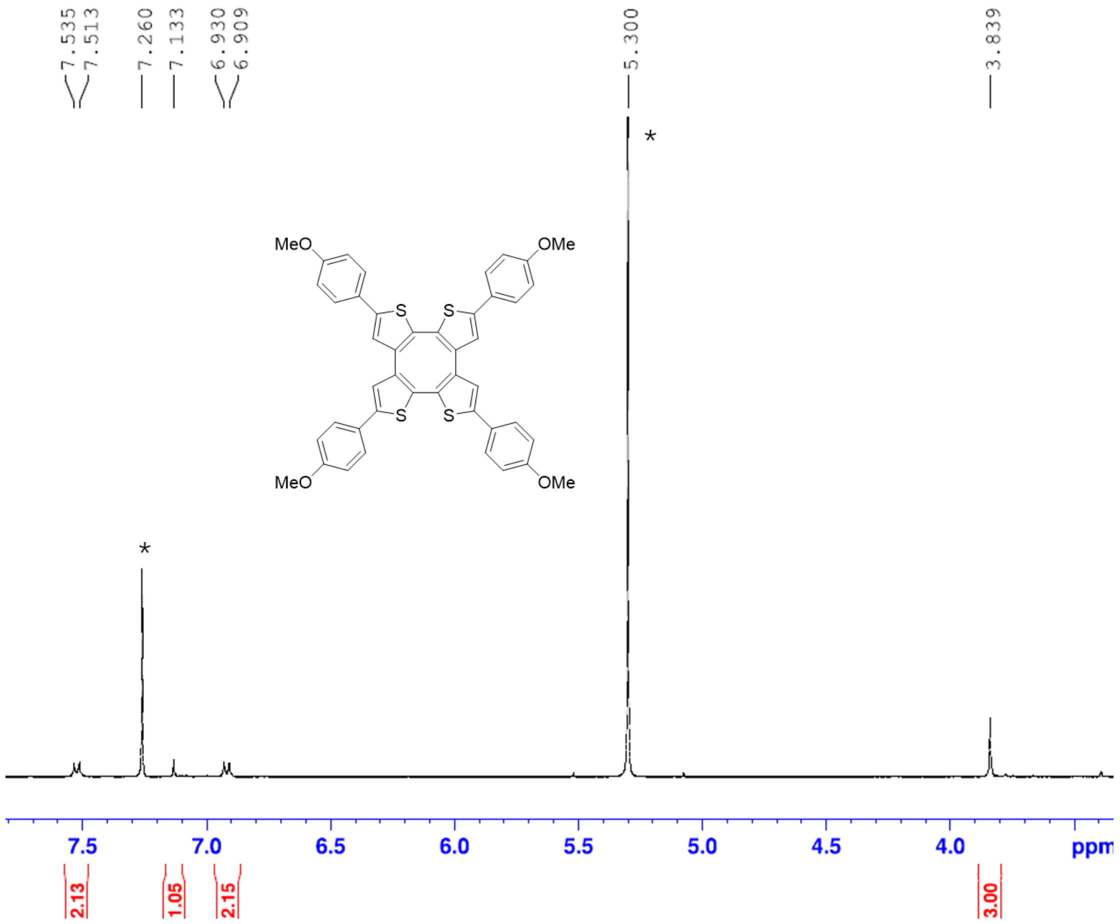

Supplementary Figure 2. ^{1}H NMR of compound **3** in $CDCl_3$ at 298K.

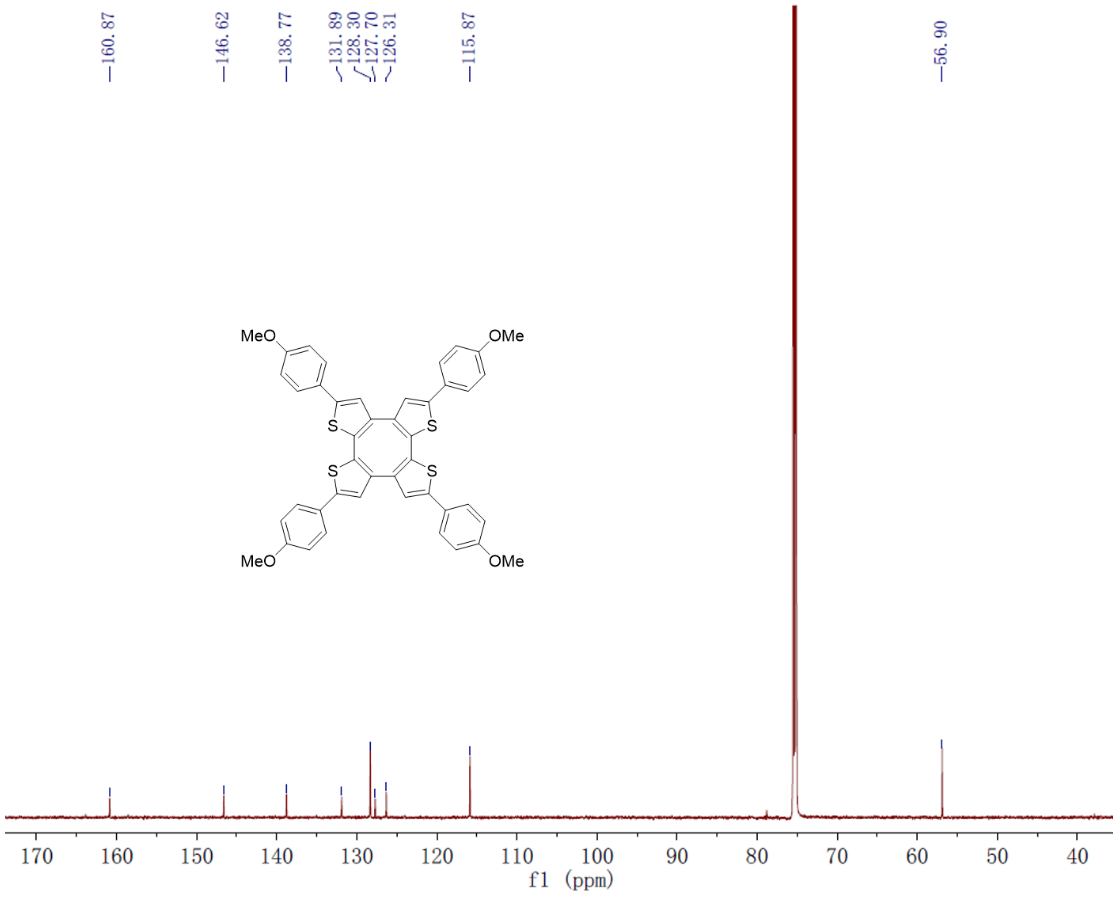

Supplementary Figure 3. ^{13}C NMR of compound **3** in 1,1,2,2-tetrachloroethane-d_2 at 298K (150 MHz).

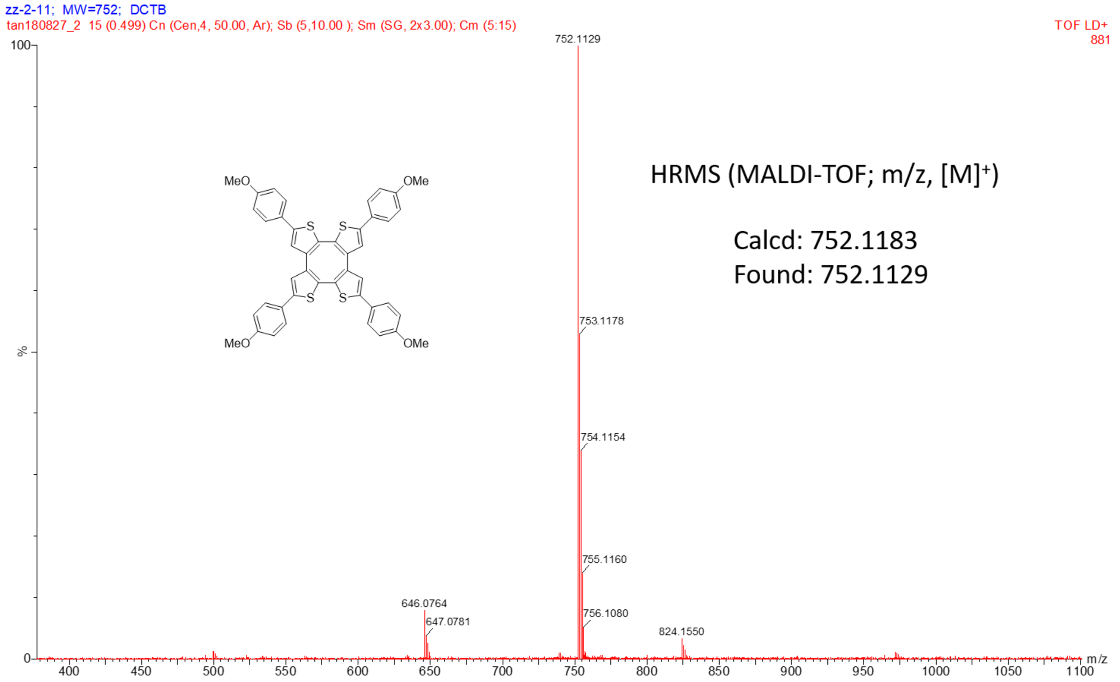

Supplementary Figure 4. High resolution mass spectrum of compound **3**.

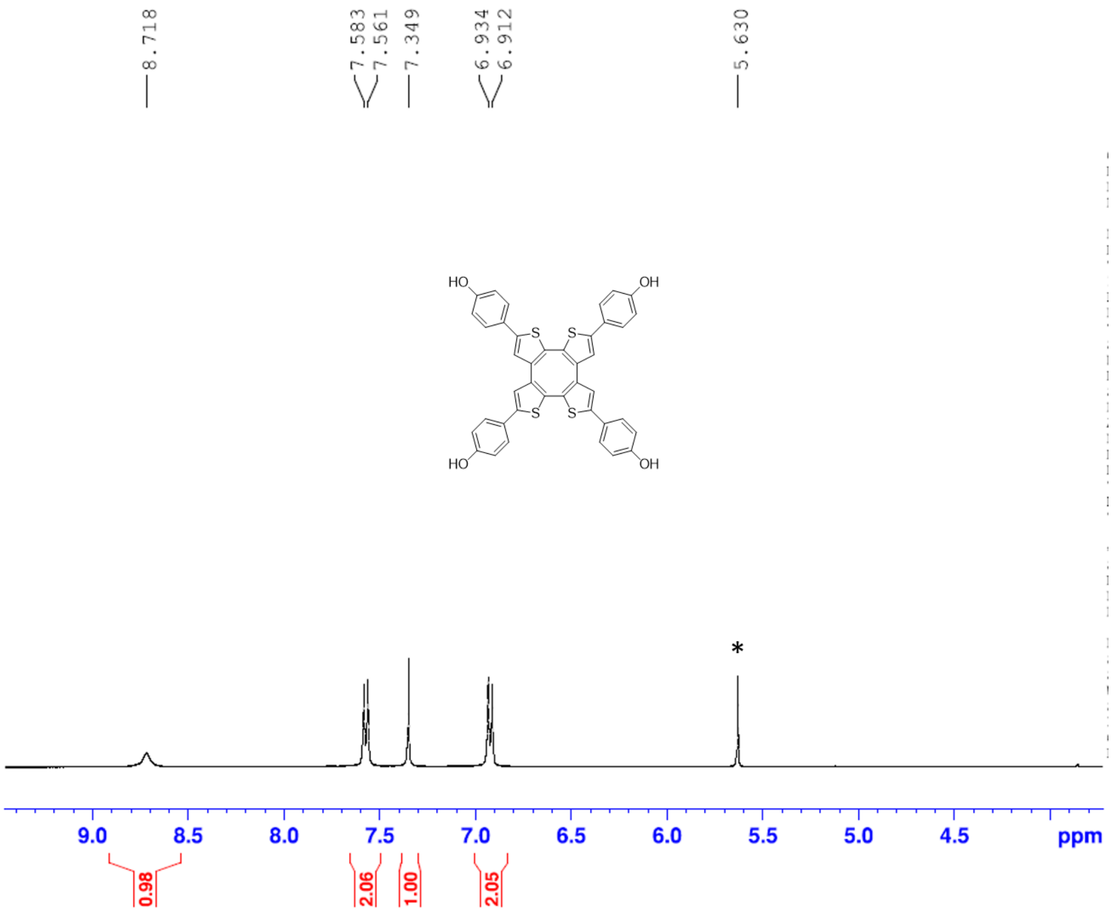

Supplementary Figure 5. ^{1}H NMR of compound **2** in $(CD_3)_2CO$ at 298K.

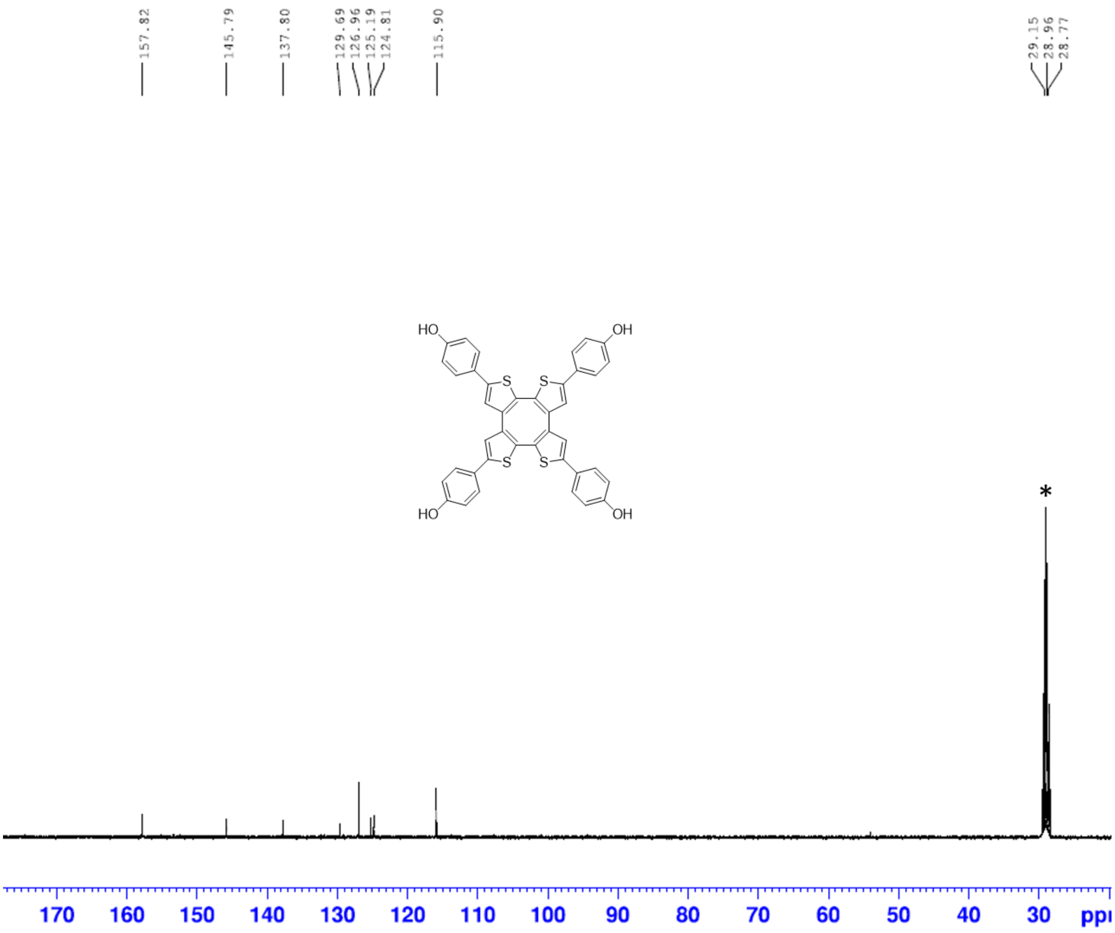

Supplementary Figure 6. ^{13}C NMR of compound **2** in $C_2D_2Cl_4$ at 298K.

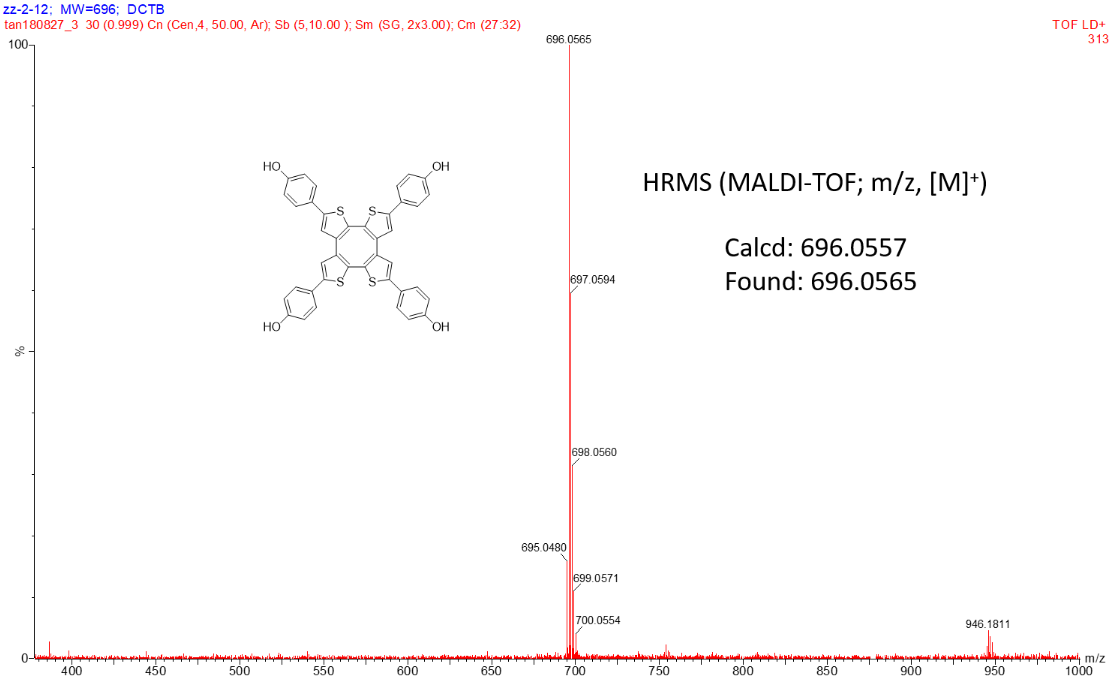

Supplementary Figure 7. High resolution mass spectrum of compound **2**.

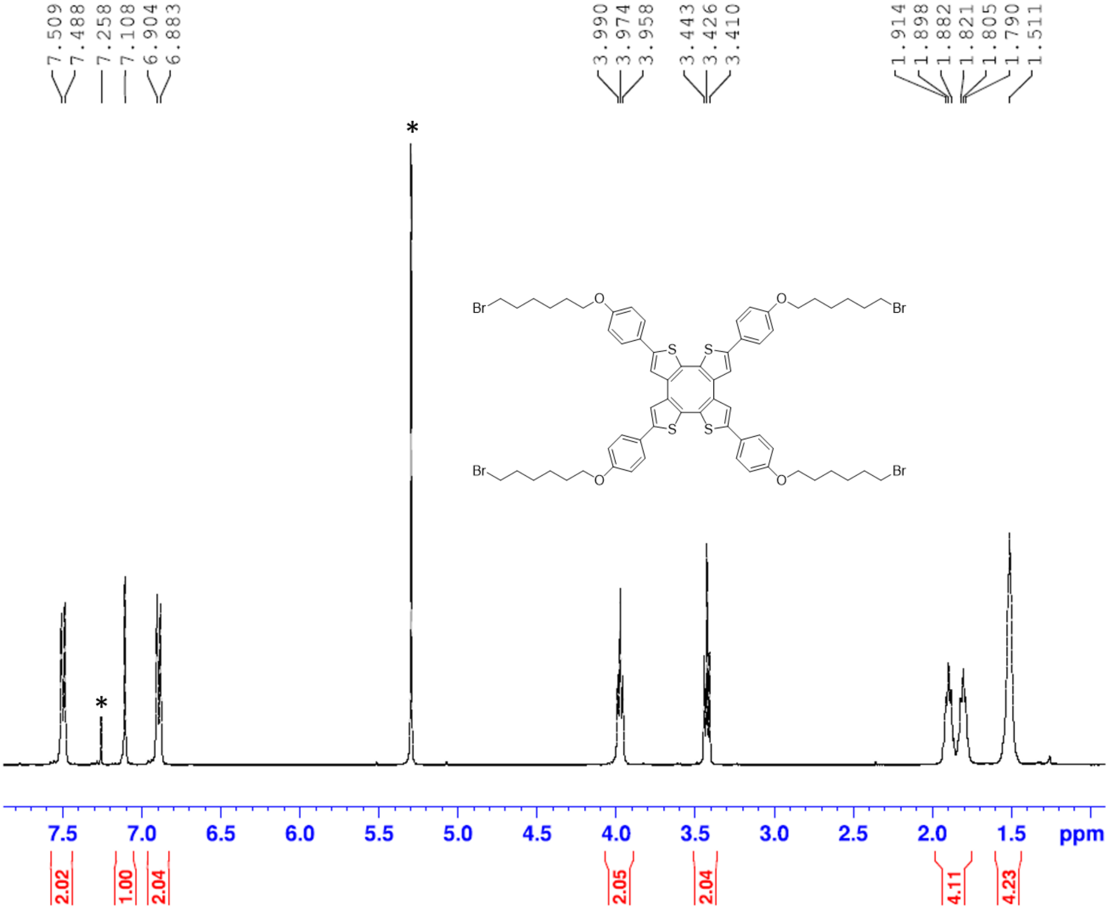

Supplementary Figure 8. ^{1}H NMR of compound **1** in $CDCl_3$ at 298K.

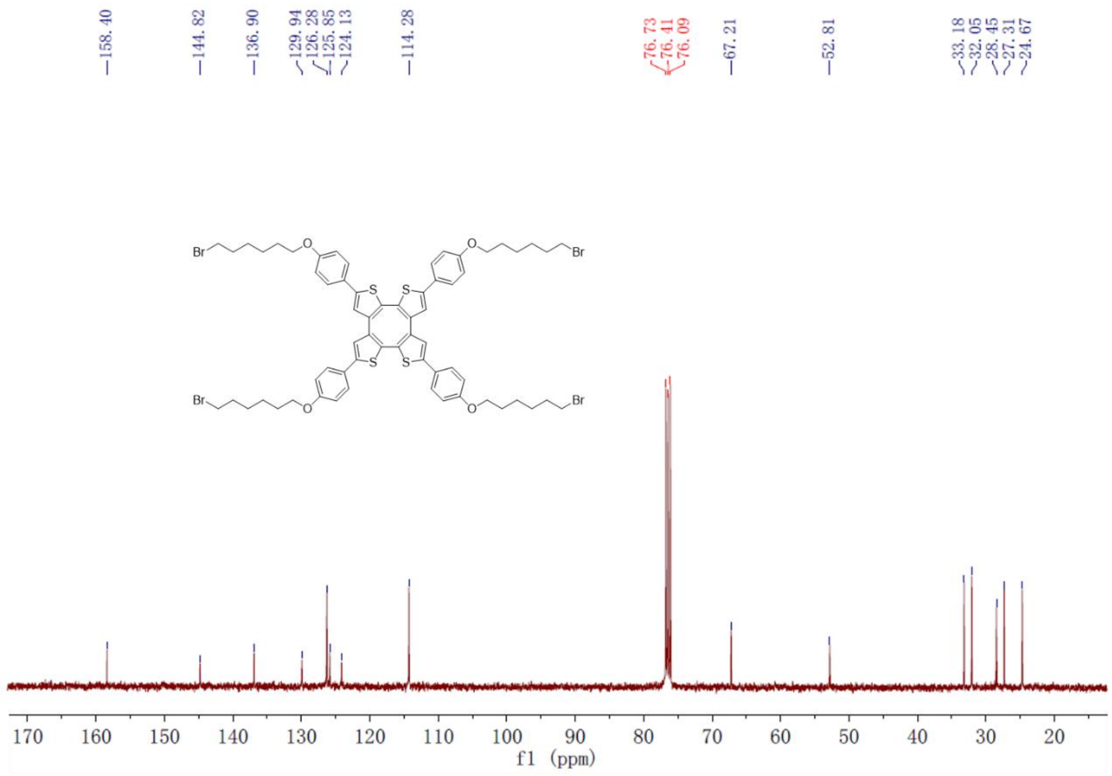

Supplementary Figure 9. ^{13}C NMR of compound **1** in $CDCl_3$ at 298K.

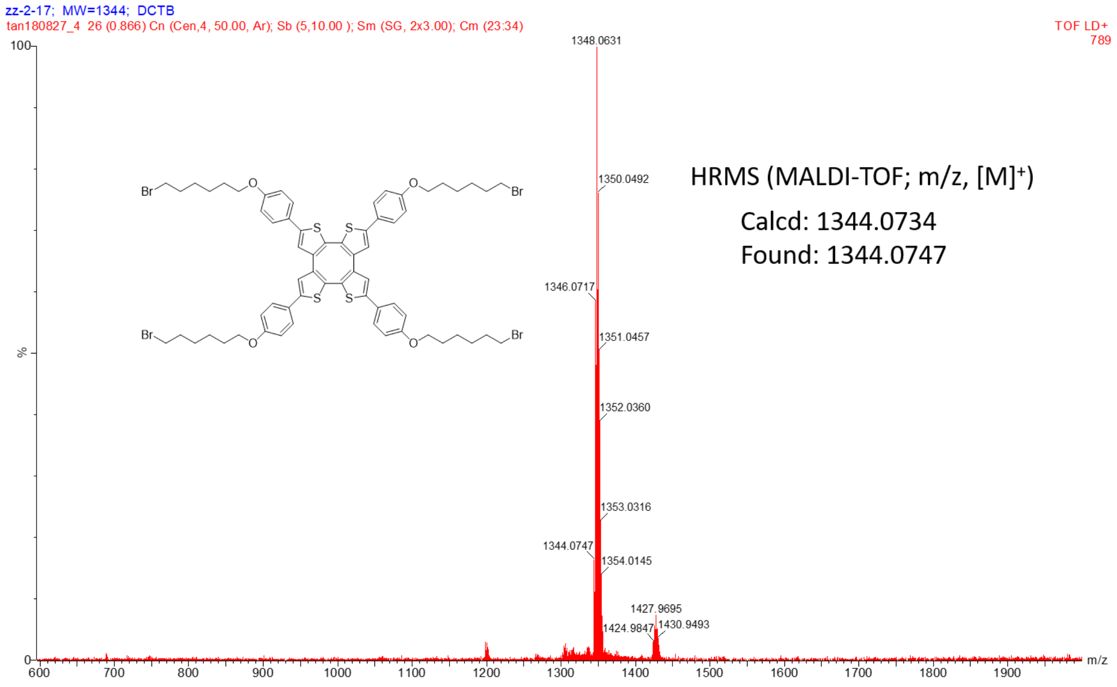

Supplementary Figure 10. High resolution mass spectrum of compound **1**.

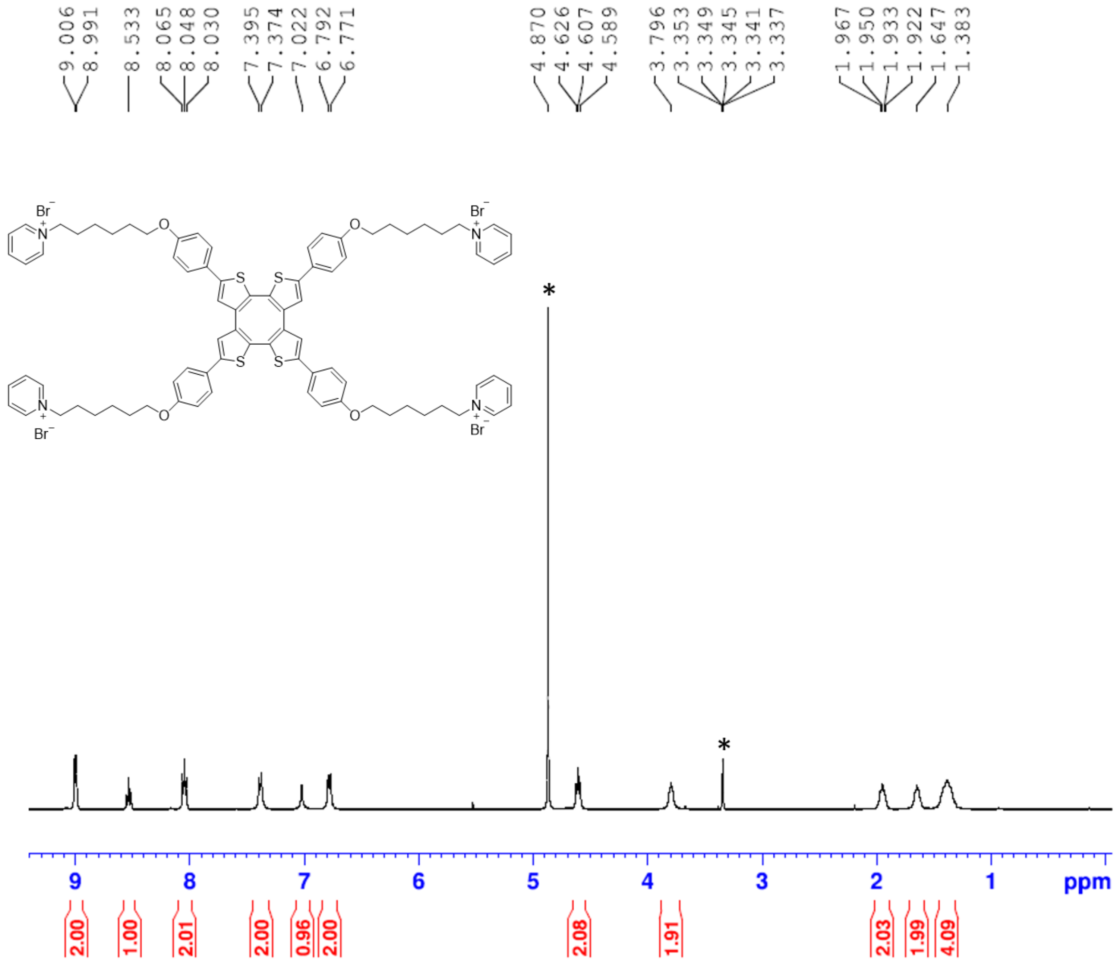

Supplementary Figure 11. ^{1}H NMR of compound **COTh-Py** in CD_3OD at 298K.

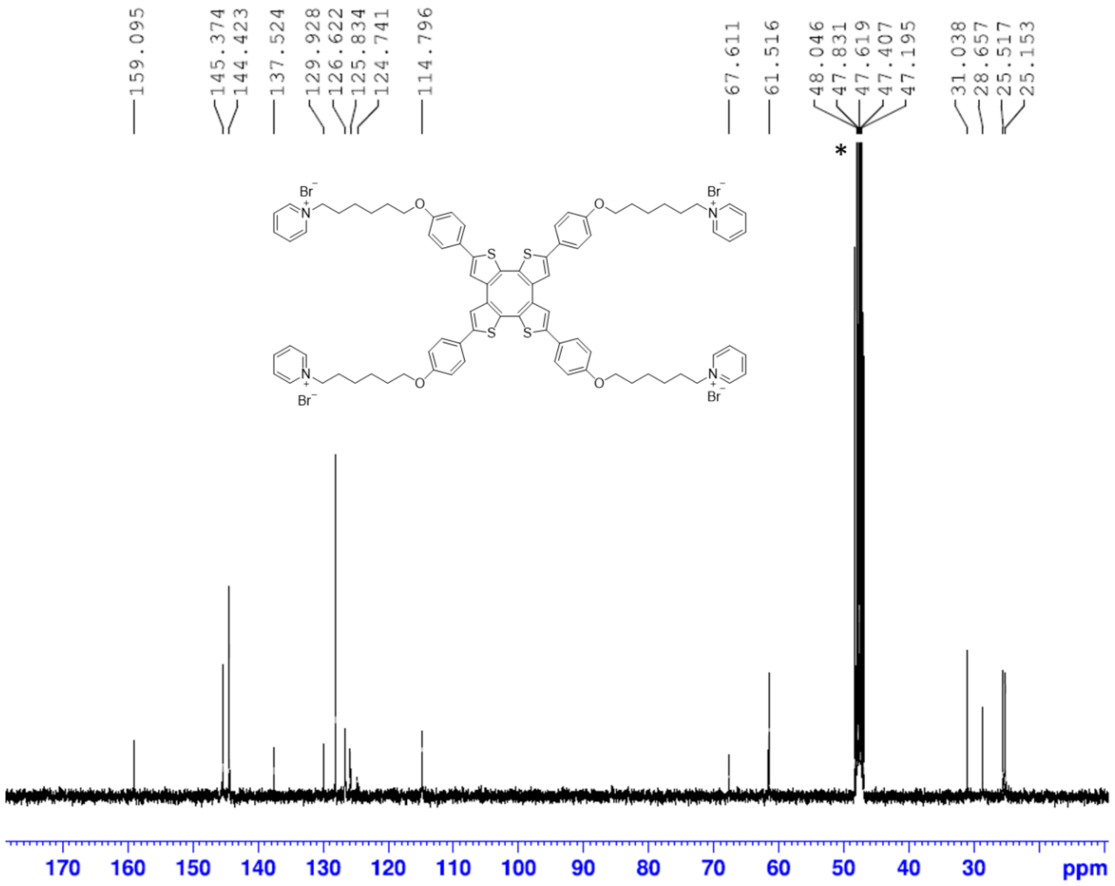

Supplementary Figure 12. ^{13}C NMR of compound **COTh-Py** in CD_3OD at 298K.

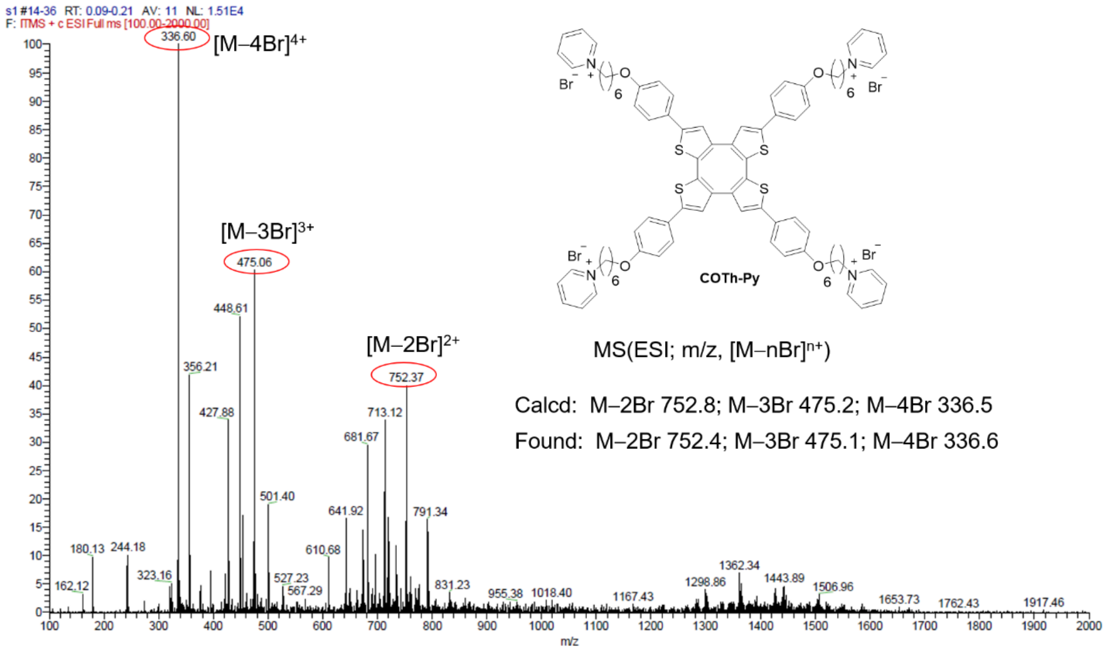

Supplementary Figure 13. Mass spectrum of compound **COTh-Py**.

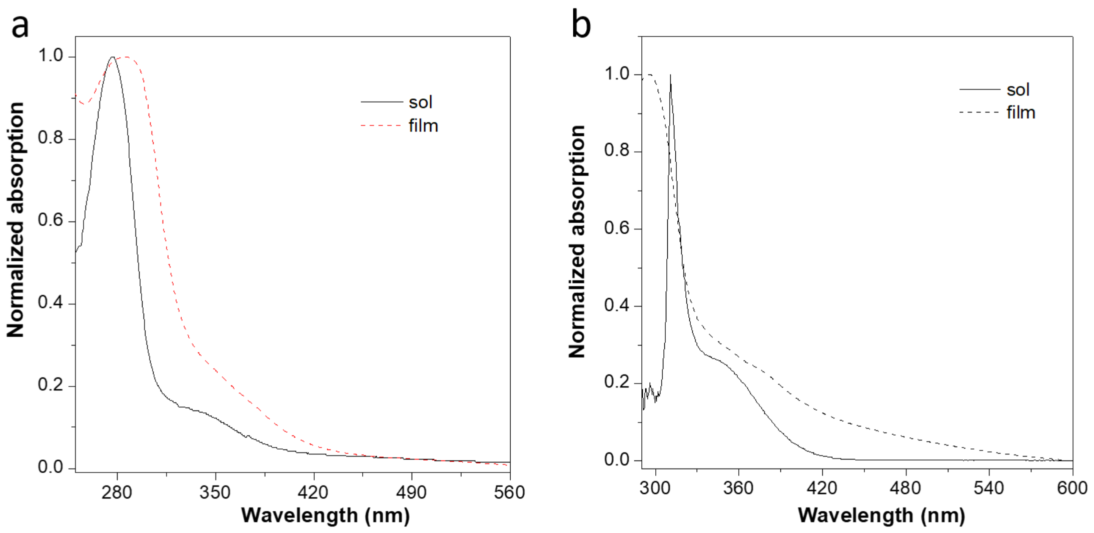

Supplementary Figure 14. The UV spectra of COTh system. a, The absorption of COTh in THF solution and thin film. **b,** The absorption of COTh-TMS in THF solution and thin film.

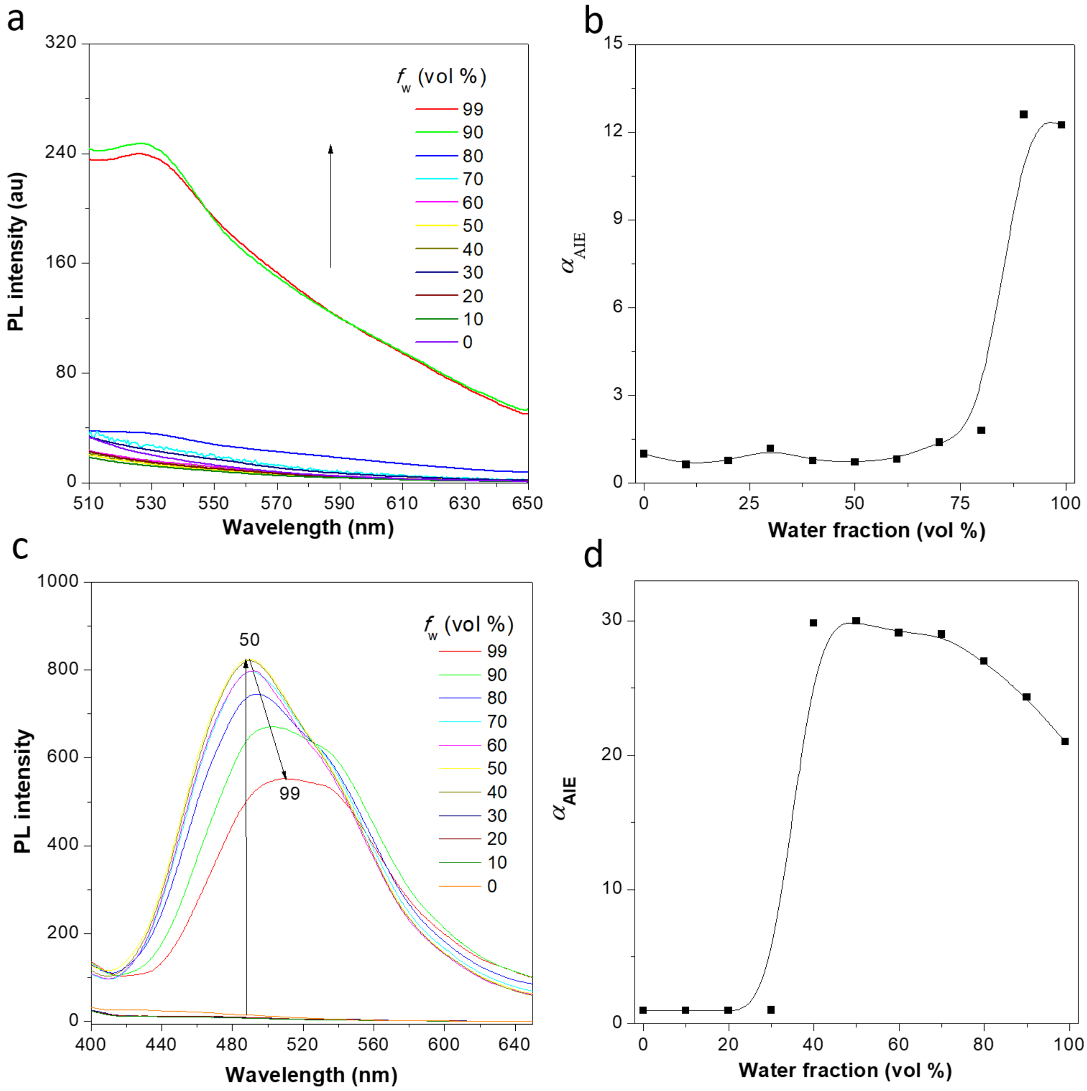

Supplementary Figure 15. Photoluminescence properties of COTh and COTh-TMS. **a,** Fluorescence emission spectra of COTh with different water fraction in acetone/water mixtures**. b**, The plots of maximum emission intensity of COTh versus water fraction in acetone/water mixtures. **c,** Fluorescence emission spectra of COTh-TMS with different water fraction in acetone/water mixtures**. d**, The plots of maximum emission intensity of COTh-TMS versus water fraction in acetone/water mixtures. Excitation wavelength: 360 nm, concentration of COTh and COTh-TMS: 10.0 μM.

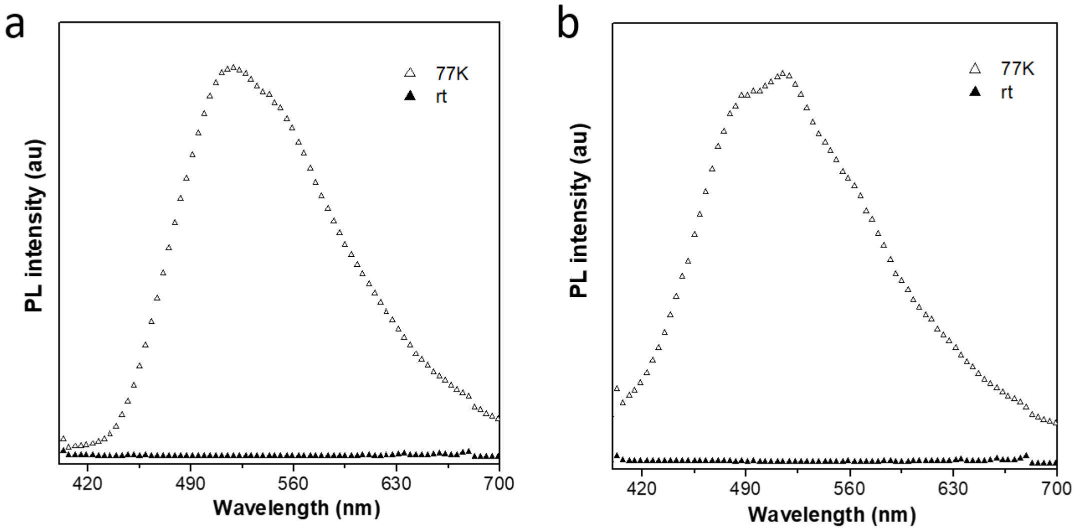

Supplementary Figure 16. Fluorescence emission spectra at room temperature and 77k. **a**, Emission of the solution of COTh at room temperature and 77k. **b**, Emission of the solution of COTh-TMS at room temperature and 77k.

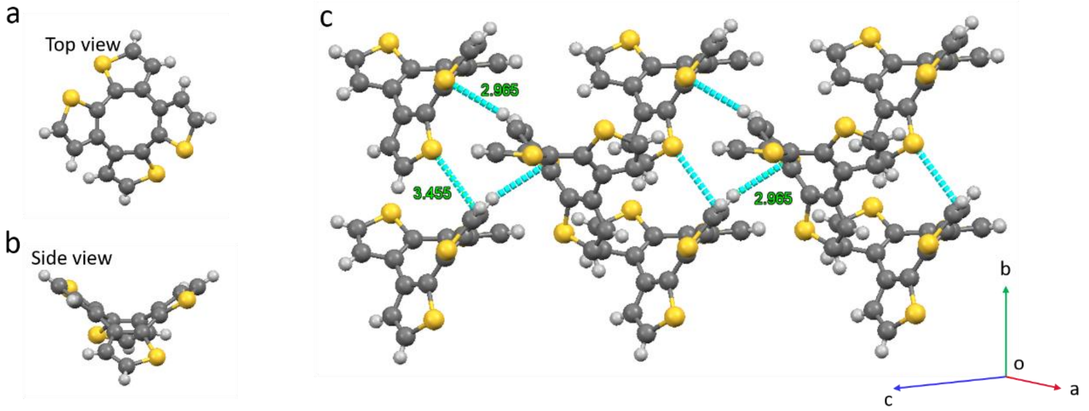

Supplementary Figure 17. Single crystal structure of COTh. a, Top view. **b**, Side view. **c**, Intermolecular interactions (S…π: 3.455Å, C-H…S: 2.965Å).

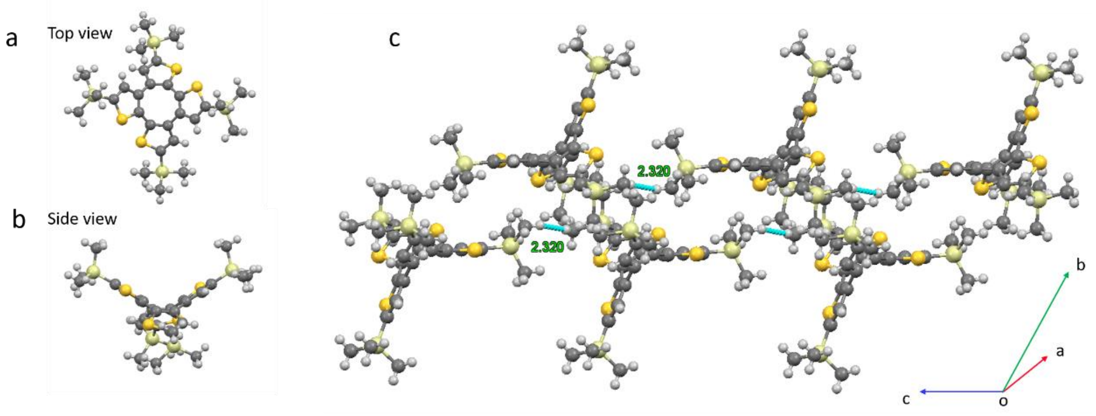

Supplementary Figure 18. Single crystal structure of COTh-TMS. a, Top view. **b**, Side view. **c–d**, Intermolecular interactions (Si-H…H-Si: 2.336Å, 2.395Å, 2.320Å).

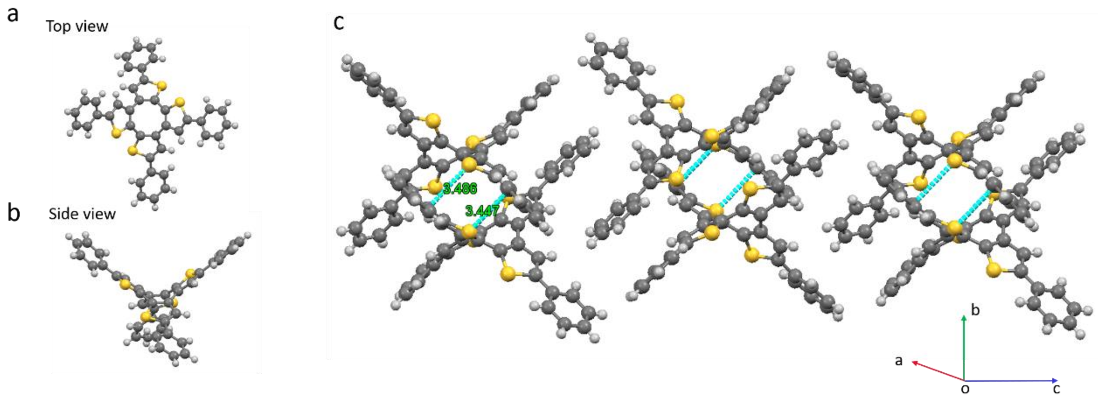

Supplementary Figure 19. Single crystal structure of COTh-Ph. a, Top view. **b,** Side view. **c–e,** Intermolecular interactions (S…π: 2.878Å, 3.447Å, 3.486Å).

Computational details.

Electronic structures. The geometry optimization and frequency calculation were carried out for the ground state (S_0) at the DFT/B3LYP/SV(P) level and for the first excited singlet state (S_1) at TDDFT/B3LYP/SV(P) level respectively, for COTh molecule at both gas phase and crystal state. Here, all these calcualtion were preformed with Turbomole 6.5 program[3]. The solid state effect were mimicked through a combined quantum mechanics and molecular mechanics (QM/MM) by extracting a supercell from the experimental crystal structure, where the central molecule is selected as the QM region and its surrondings are taken as the MM region (Supplementary Figure 10). The atom types of COTh molecule in MM region are built based on the general amber force field (GAFF)[4]. The QM/MM was interfaced by using the ChemShell 3.5 package[5], combing Turbomole 6.5 and DLPOLY programs[1] to obtain the QM/MM energy and energy gradient of the QM/MM. The interaction between the QM and MM parts is through electrostatic Hamiltonian[7]. The electrostatic interactions were dealt with embedding scheme in the QM/MM calculations[2-3]. Explicitely, the partial charge of each atom in the MM region was combined into one-electon part of QM Hamiltonian, then the electrostatic interactions between QM and MM part is evaluated by the electrostatic potential of two parts. Therefore, the QM region can reflect all the electonic properties while MM region consider the environment effects. During the geometric optimizaton process, all atoms in MM region were frozen, only atoms in the QM region are free to relax. The numerical two-point displacement method is chosen to calculate the vibrational frequency and the ploarization effect of environment was included. Here, the absence of imargnary frequency for all geometries for both ground and excited state were carefully checked. No symmetric constraints were imposed during the geometric optimization.

Radiative and non-radiative decay rate constants calculations. The radiative decay rate constant (k_r) was obtained by integrating over the whole emission spectra:

$$k_r(T) = \int \sigma_{em}(\omega, T) d\omega$$

(1)

$$\sigma_{em}(\omega,T)=\frac{4\omega^3}{3\hbar c^3}\sum_{u,v}P_{iv}(T)\left|\langle\Theta_{f\mathrm{u}}\right|\vec{\mu}_{fi}\left|\Theta_{iv}\rangle\right|^2\delta(\omega_{iv,fu}-\omega)$$

(2)

Here P_{iv} represents the Boltzmann distribution function of the initial state at specific temperature. Θ is the nuclear vibrational wave function. $\vec{\mu}_{fi}=\langle\Phi_f|\vec{\mu}|\ \Phi_i\rangle$ is the electric transition dipole moment between two electronic states.

Based on the Fermi Golden Rule (FGR), the non-radiative internal conversion (IC) decay rate constant can be written as[9]:

$$k_{IC}=\frac{2\pi}{\hbar}\sum_{u,v}P_{iv}\left|\hat{\mathrm{H}}'_{\mathrm{fu},iv}\right|^2\delta(E_{iv}-E_{fu})$$

(3)

Here, $E_{fv}(E_{fu})$ reflects the electronic and vibrational energy of the initial (final) state, $\hat{H}'$ represents the non-Born-Oppenheimer coupling.

Based on the Franck-Condon principle, applying Fourier transform of the δ-function, equation (3) can be written as[4-5]:

$$k_{IC}=\sum_{kl}\frac{1}{\hbar^2}R_{kl}\int_{-\infty}^{\infty}\mathrm{d}t\left[e^{i\omega_{if}t}Z_i^{-1}\rho_{IC}(t,T)\right]$$

(4)

$\mathrm{R}_{kl}=\langle\Phi_f|\hat{P}_{fk}|\Phi_i\rangle\langle\Phi_i|\hat{P}_{fl}|\ \Phi_f\rangle$ is the non-adiabatic electronic coupling. $\rho_{IC}(t,T)$ is the thermal vibration correlation function (TVCF)[6-8],

$$\rho_{IC,kl}(t,T)=Tr\left(\hat{P}_{fk}e^{-i\tau_f\hat{H}_f}\hat{P}_{fl}e^{-i\tau_i\hat{H}_i}\right)$$

(5)

Based on the electronic structure information obtained from purely QM and QM/MM optimization of the isolated molecule and cluster, the radiative and non-radiative rate constants were calculated by solving Equation (1) and Equation (3) through the TVCF in the MOMAP program[95]. The difference between potential energy surfaces of the ground and excited states is considered by $Q_i=SQ_f+\mathrm{D}$, where S is Duschinsky rotation matrix and D is the displacement vector. The non-adiabatic electronic couplings

were calculated by using the exact analytical derivative couplings between the time-dependent Kohn-Sham determinants in a finite atom-centered basis set in TURBOMOLE program[10].

Nucleus-independent chemical shift (NICS) and anisotropy of the induced current density (ACID) calculations. NICS calculation was performed at DFT/B3LYP/6-31G** level and AICD plot was calculated at B3LYP/6-31G(d,p) level by using the method developed by Herges[17].

Supplementary Table 1. Selected bond lengths (in Å), bond angles (in degree), and dihedral angels (in degree) of molecule COTh at S_0/S_1 geometries and their difference in both gas phase and crystal phase.

	gas			solid			
	S_0	S_1	$\|\triangle S_0-S_1\|$	S_0	S_1	$\|\triangle S_0-S_1\|$	Crystal
S_9-C_{10}	1.73	1.73	0.00	1.72	1.74	0.02	1.70
S_9-C_1	1.75	1.78	0.03	1.75	1.77	0.02	1.73
C_1-C_8	1.46	1.41	0.05	1.46	1.41	0.05	1.46
C_3-C_2	1.48	1.43	0.05	1.48	1.43	0.05	1.47
C_5-C_4	1.46	1.41	0.05	1.46	1.41	0.05	1.46
C_7-C_6	1.48	1.43	0.05	1.48	1.43	0.05	1.47
C_1-C_2	1.39	1.44	0.06	1.39	1.44	0.05	1.37
C_3-C_4	1.39	1.44	0.06	1.39	1.44	0.05	1.37
C_5-C_6	1.39	1.44	0.06	1.39	1.44	0.05	1.37
C_7-C_8	1.39	1.44	0.06	1.39	1.44	0.05	1.37
C_{10}-S_9-C_1	92.05	92.15	0.10	92.04	91.77	0.27	92.35
C_8-S_{20}-C_{19}	92.05	92.15	0.10	92.23	91.98	0.25	91.68
C_{16}-S_{15}-C_5	92.05	92.15	0.10	92.33	92.18	0.15	92.23
C_4-S_{14}-C_{13}	92.05	92.15	0.10	92.03	91.68	0.35	91.73
C_2-C_1-C_8	129.71	133.35	3.64	129.52	129.98	0.46	129.89
C_8-C_7-C_6	125.45	129.52	4.07	125.22	126.9	1.68	125.48
C_6-C_5-C_4	129.71	129.51	0.20	129.92	130.26	0.34	129.96

C_4-C_3-C_2	125.45	129.51	4.06	125.35	127.04	1.69	124.71
C_3-C_2-C_1-C_8 (Φ_I)	0.25	1.37	1.12	1.99	5.272	3.28	1.20
C_5-C_4-C_3-C_2 (Φ_{II})	0.24	1.37	1.13	-0.44	-1.65	1.21	0.83
C_7-C_6-C_5-C_4 (Φ_{III})	0.25	1.37	1.12	2.16	5.94	3.78	4.15
C_1-C_8-C_7-C_6 (Φ_{IV})	0.24	1.37	1.13	-0.58	-2.738	2.16	-1.2
C_{12}-C_3-C_2-C_{11} ($\Theta_{I\text{-}II}$)	44.93	22.11	22.82	43.84	31.06	12.78	42.85
S_{15}-C_5-C_4-S_{14} ($\Theta_{II\text{-}III}$)	-48.58	-24.36	24.22	-48.67	-35.71	12.96	-48.83
C_{18}-C_7-C_6-C_{17} ($\Theta_{III\text{-}IV}$)	44.92	22.11	22.81	45.24	32.6	12.64	45.16
S_9-C_1-C_8-S_{20} ($\Theta_{I\text{-}IV}$)	-48.59	-24.36	24.23	-48.94	-35.72	13.22	-46.99

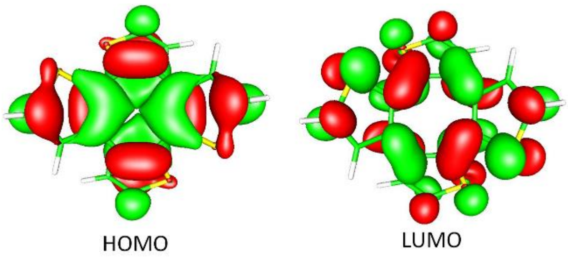

Supplementary Figure 20. The electron density distribution of molecular orbitals HOMO and LUMO of COTh molecule at the top view.

Supplementary Table 2. Calculated important parameters related to the emission spectrum.

	ΔE (eV)	*f*	EDM/D	assignment (H→L)
gas phase	2.42	0.002	0.477	99.5%
solid phase	2.69	0.003	0.645	99.3%

Supplementary Table 3. Calculated radiative rate (k_r), non-radiative rate (k_{ic}) and the quantum yield (η_F).

	k_r (10^6 s^{-1})	k_{ic} (10^6 s^{-1})	η_F (%)
gas phase	0.098	4130	0.002

solid phase	0.605	18.7	3.13

Supplementary Table 4. Projection the reorganization energy of COTh molecule into internal coordinates: bond length, bond angle and dihedral angle, respectively (unit: meV)

	bond length	bond angle	dihedral angle
gas	315.4	15	529.6
solid	312.83	28.6	294.6

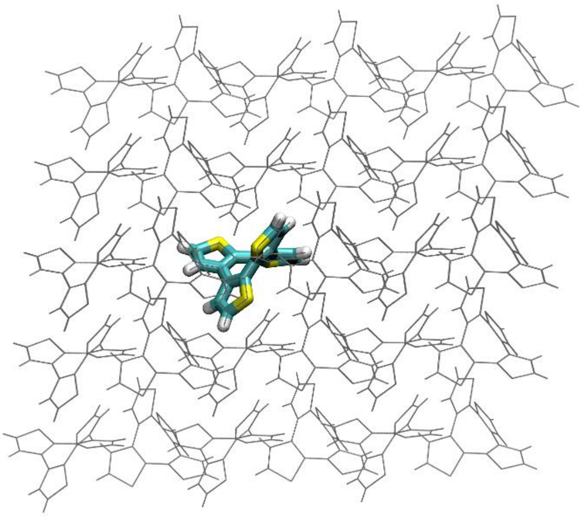

Supplementary Figure 21. The QM/MM model of COTh molecule. The single COTh molecule in cluster center is treated as QM part to obtain the electronic information for both ground and excited state. All the other 53 molecules surrounded are taken as the MM part to mimicked the environment of the solid state.

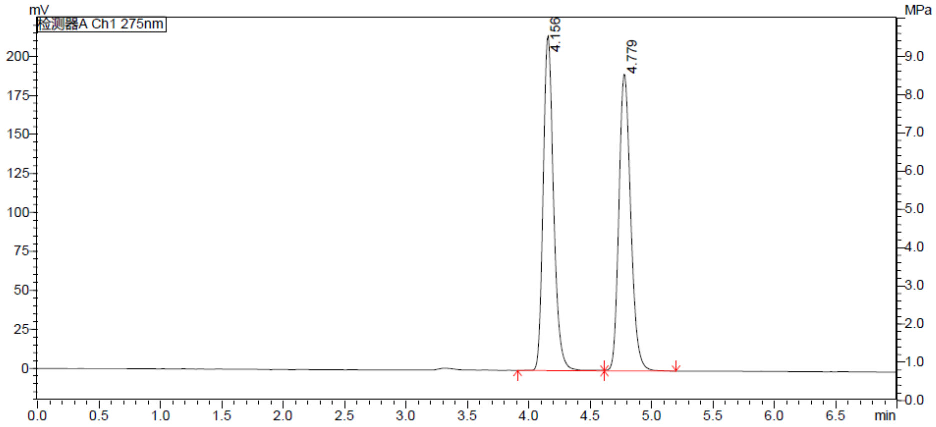

<Column Performance Report>

Peak#	Ret. Time	Area	Area%	T.Plate#	Tailing F.	Resolution
1	4.156	1268269	49.890	10006	1.241	--
2	4.779	1273865	50.110	10469	1.203	3.529

Supplementary Figure 22. The HPLC spectrum of the racemic mixture of COTh before chiral resolution .

<Chromatogram>

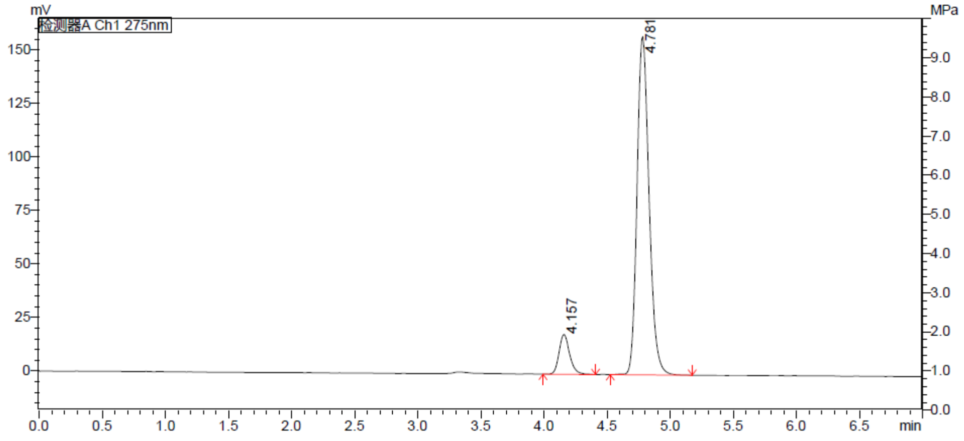

<Column Performance Report>

Peak#	Ret. Time	Area	Area%	T.Plate#	Tailing F.	Resolution
1	4.157	109653	9.455	10116	1.224	--
2	4.781	1050108	90.545	10652	1.198	3.557

Supplementary Figure 23. The HPLC spectrum of enantiomer COTh (+) after chrial resolution.

<Chromatogram>

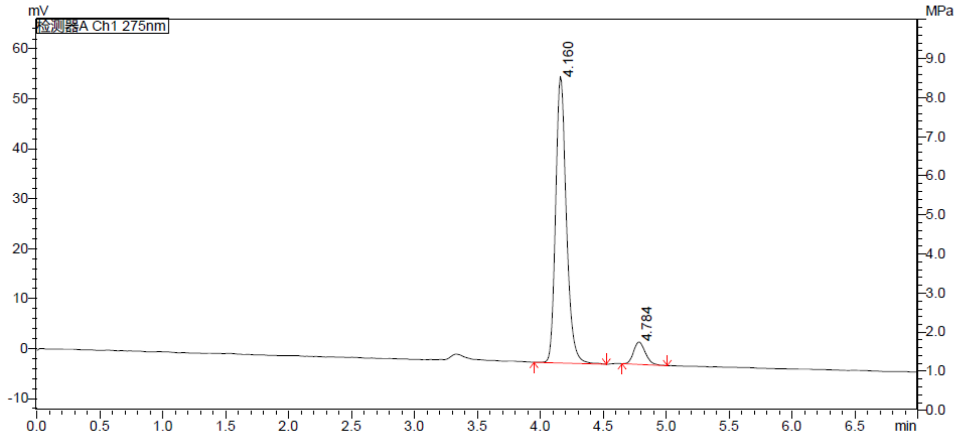

<Column Performance Report>

Peak#	Ret. Time	Area	Area%	T.Plate#	Tailing F.	Resolution
1	4.160	340779	92.157	9933	1.238	--
2	4.784	29000	7.843	10933	1.194	3.566

Supplementary Figure 24. The HPLC spectrum of enantiomer COTh (−) after chiral resolution.

Supplementary Table 5. Crystal data and structure refinement of COTh (+).

Identification code	COTh (+)
Empirical formula	$C_{16}H_8S_4$
Formula weight	328.46
Temperature/K	99.96(10)
Crystal system	monoclinic
Space group	$P2_1$
a/Å	9.5173(3)
b/Å	7.1942(2)
c/Å	10.2556(3)
α/°	90
β/°	104.186(3)
γ/°	90
Volume/Å^3	680.78(4)
Z	2
ρ_{calc} g cm^{-3}	1.602
μ/mm^{-1}	6.264
F(000)	336.0
Crystal size/mm^3	0.08 × 0.05 × 0.04
Radiation	CuKα (λ = 1.54184)
2Θ range for data collection/°	8.894 to 134.972
Index ranges	$-11 \leq h \leq 11$, $-8 \leq k \leq 8$, $-8 \leq l \leq 12$
Reflections collected	3705
Independent reflections	2364 [R_{int} = 0.0164, R_{sigma} = 0.0249]
Data/restraints/parameters	2364/1/181
Goodness-of-fit on F^2	1.004

Final R indexes [I>=2σ (I)]	R_1 = 0.0222, wR_2 = 0.0563
Final R indexes [all data]	R_1 = 0.0226, wR_2 = 0.0569
Largest diff. peak/hole / e $Å^{-3}$	0.26/-0.18
Flack parameter	0.015(12)

Supplementary Table 6. Crystal data and structure refinement for COTh (-).

Identification code	COTh (-)
Empirical formula	$C_{16}H_8S_4$
Formula weight	328.46
Temperature/K	100.01(10)
Crystal system	monoclinic
Space group	$P2_1$
a/Å	9.5152(3)
b/Å	7.1962(2)
c/Å	10.2472(4)
α/°	90
β/°	104.131(4)
γ/°	90
Volume/$Å^3$	680.43(4)
Z	2
ρ_{calc}g cm^{-3}	1.603
μ/mm^{-1}	6.268
F(000)	336.0
Crystal size/mm^3	0.12 × 0.1 × 0.05
Radiation	CuKα (λ = 1.54184)
2Θ range for data collection/°	8.898 to 134.976

Index ranges	$-11 \leq h \leq 11, -6 \leq k \leq 8, -12 \leq l \leq 12$
Reflections collected	3602
Independent reflections	1965 [R_{int} = 0.0177, R_{sigma} = 0.0204]
Data/restraints/parameters	1965/1/181
Goodness-of-fit on F^2	1.016
Final R indexes [I>=2σ (I)]	R_1 = 0.0206, wR_2 = 0.0528
Final R indexes [all data]	R_1 = 0.0209, wR_2 = 0.0532
Largest diff. peak/hole / e Å^{-3}	0.40/-0.19
Flack parameter	0.048(13)

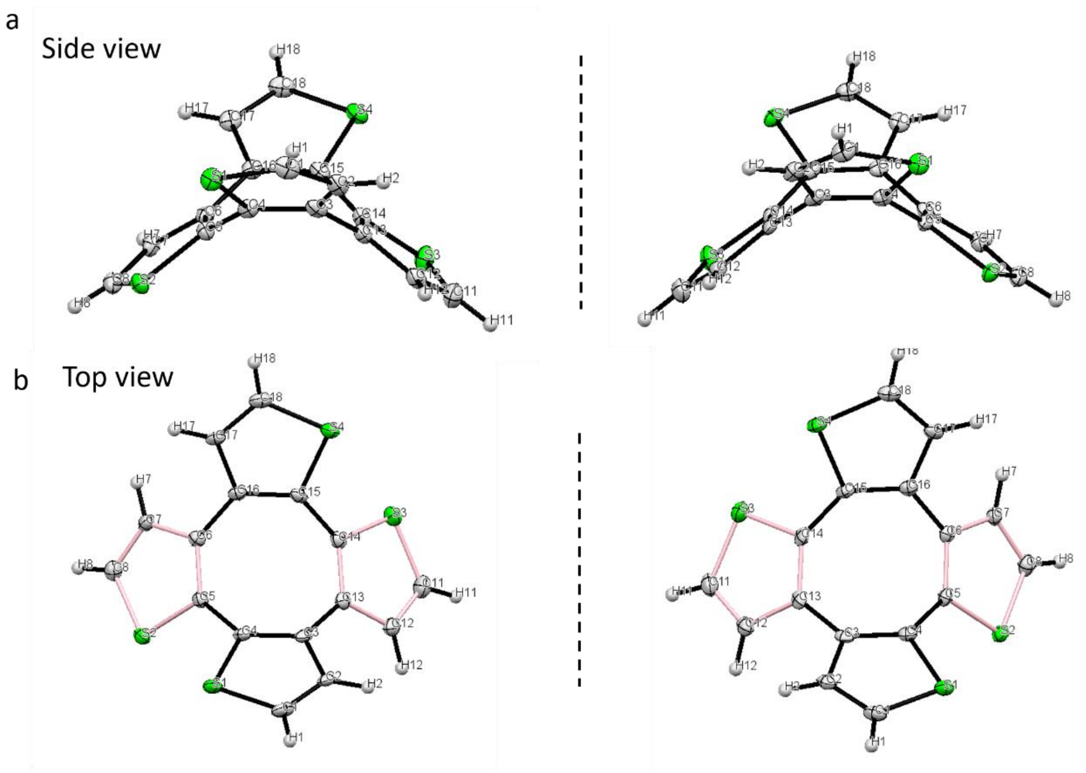

Supplementary Figure 25. The crystal structures of the enantiomers. **a**. Side view of the two enantiomers (COTh (+) left, COTh (–) right). **b**. Top view of the two enantiomers (COTh (+) left, COTh (–) right).

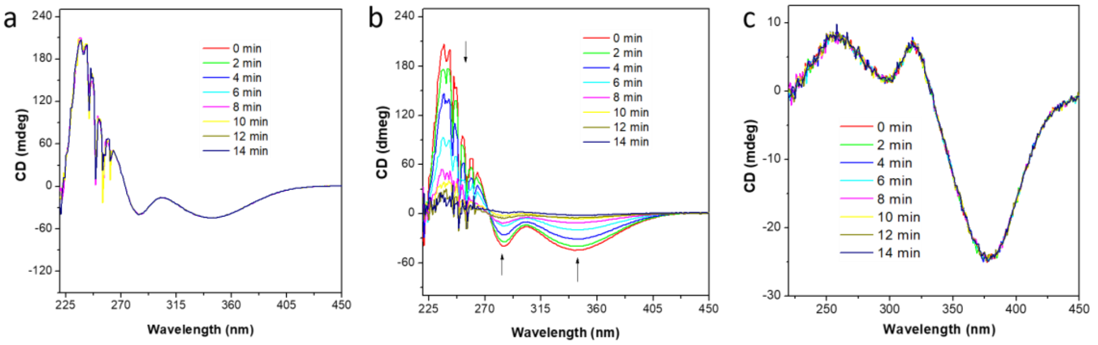

Supplementary Figure 26. Circular dichromism (CD) Spectrascopy of COTh (−). **a**, The CD spectra degradation of enantiomers of COTh (−) in THF solution without UV irradiation. **b**, The CD spectra degradation of enantiomers of COTh (−) in THF solution with UV irradiation. **c** The CD spectra degradation of enantiomers of COTh (−) in solid state with UV irradiation. The irradiation wavelength of UV lamp: 254 nm.

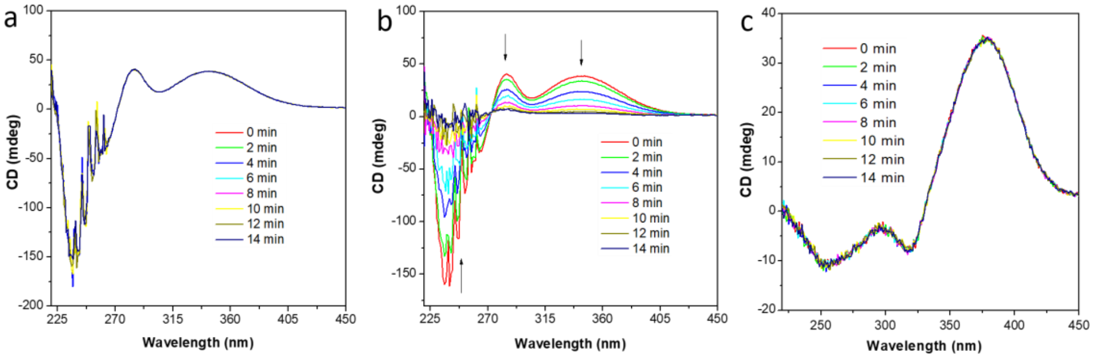

Supplementary Figure 27. Circular dichromism (CD) Spectrascopy of COTh (+). **a**, The CD spectra degradation of enantiomers of COTh (+) in THF solution without UV irradiation. **b**, The CD spectra degradation of enantiomers of COTh (+) in THF solution with UV irradiation. **c** The CD spectra degradation of enantiomers of COTh (+) in solid state with UV irradiation. The irradiation wavelength of UV lamp: 254 nm.

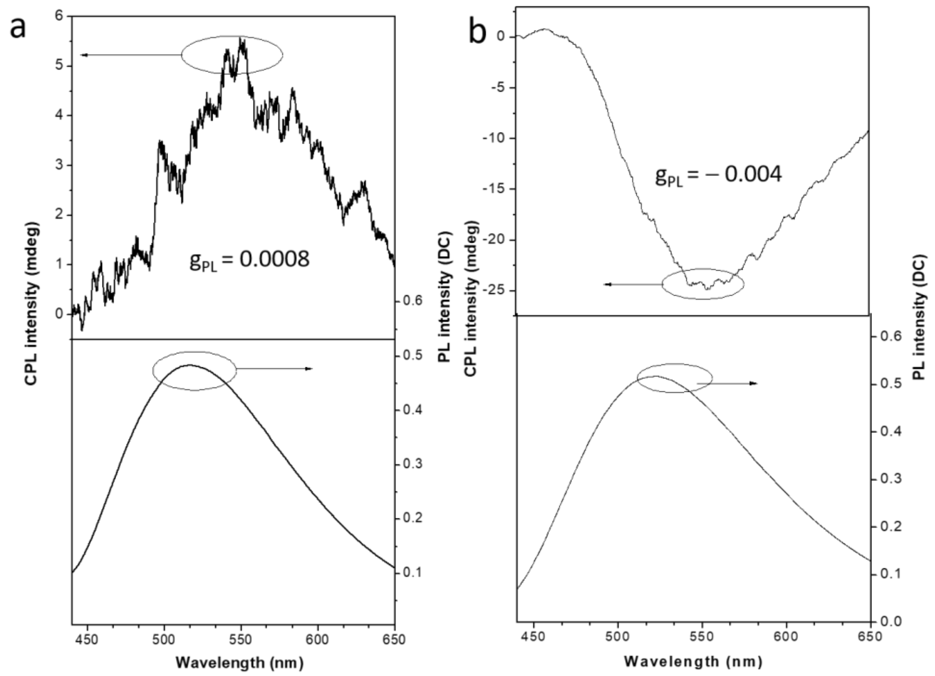

Supplementary Figure 28. CPL spectra of COTh (+) and COTh (-) in crystal. **a**. CPL spectra of COTh (+). **b**. CPL spectra of COTh (+). Excitation wavelength: 360 nm.

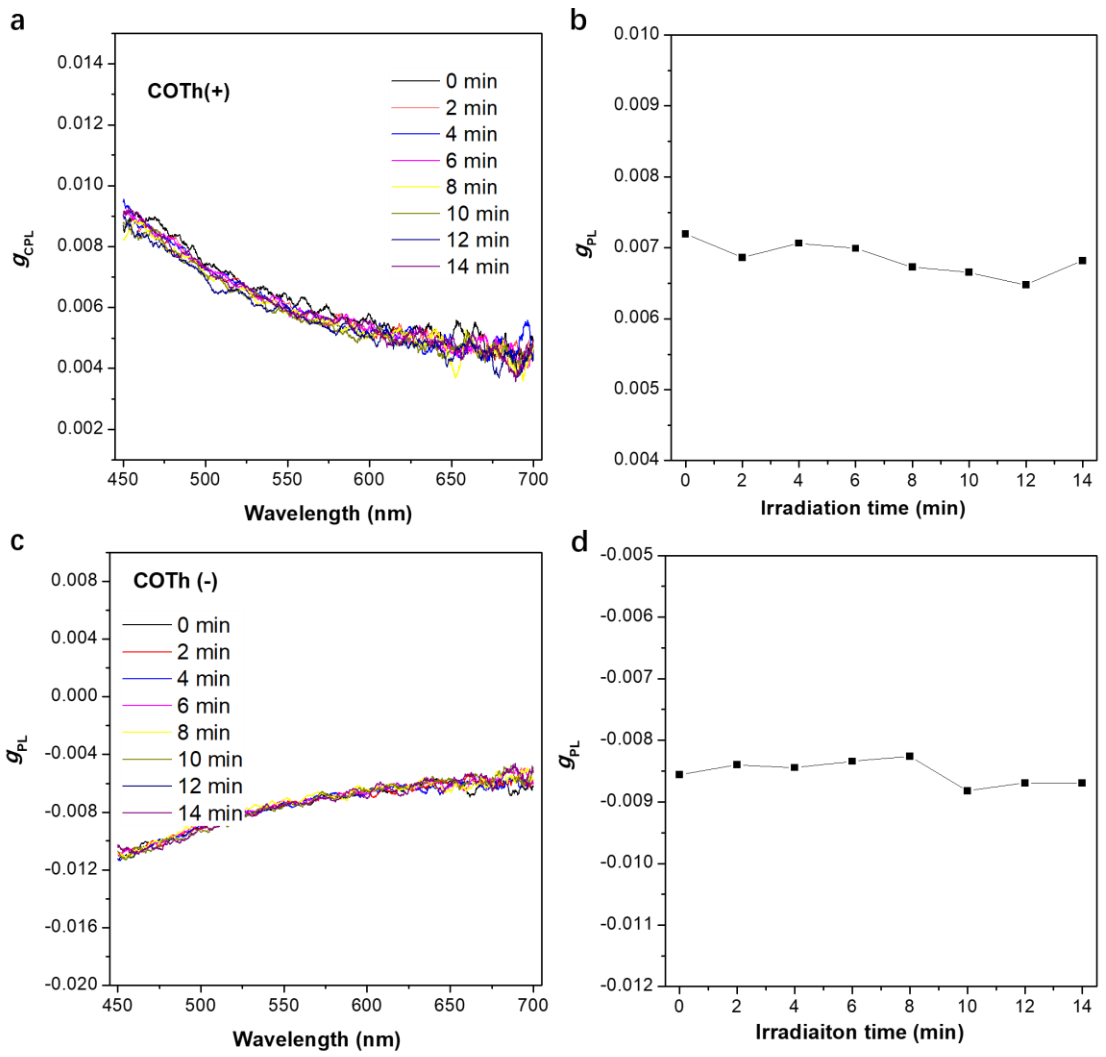

Supplementary Figure 29. g_{PL} signal of COTh (+) and COTh (–) in solid state upon UV lamp irradiation. **a**. g_{PL} spectra of COTh (+) under UV irradiaiton. **b**. The plot of g_{PL} value of COTh (+) versus irradiation time. **c**. g_{PL} spectra of COTh (–) under UV irradiation. **d**. The plot of g_{PL} value of COTh (–) versus irradiation time. Excitation wavelength: 360 nm. The irradiation wavelength of UV lamp: 254 nm.

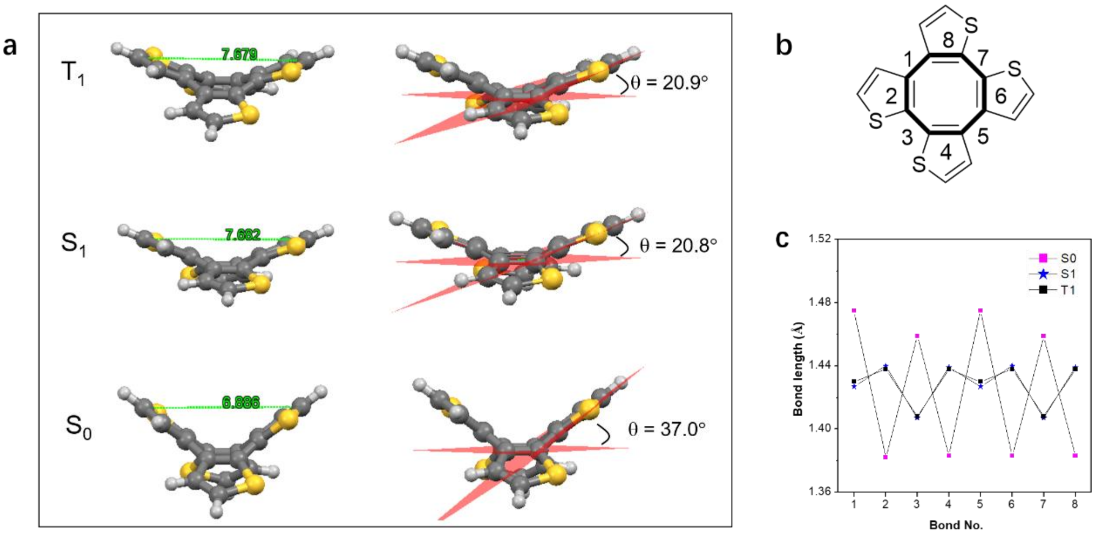

Supplementary Figure 30. The optimized minimum energy structure and the bond lengths of COTh in S_0, S_1 and T_1. a. The optimized molecular structures of COTh in S_0, S_1 and T_1. **b.** The chemical structure of COTh with bonds in the central ring labeled. **c.** Bond lengths of the central ring of COTh in S_0, S_1 and T_1.

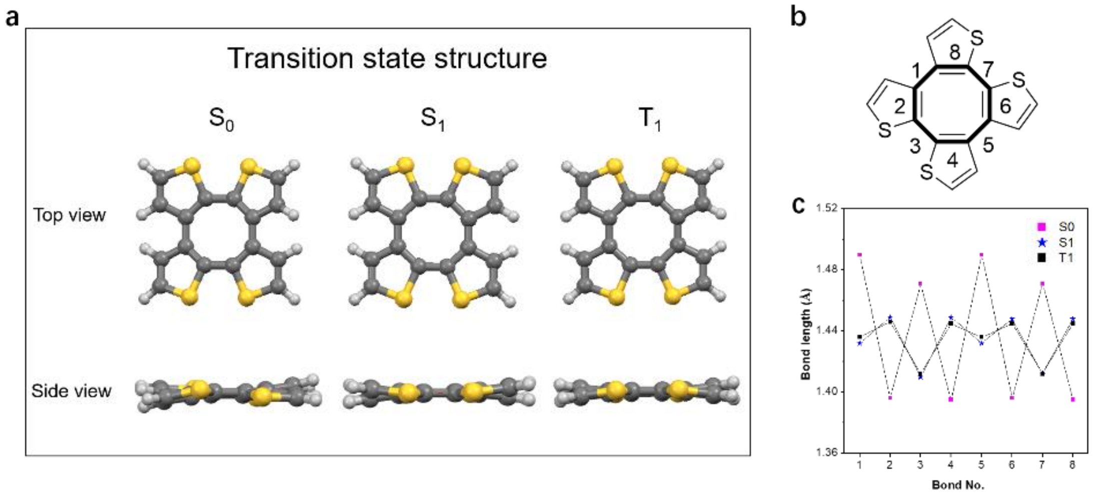

Supplementary Figure 31. The optimized transition state structure and the bond lengths of COTh in S_0, S_1 and T_1. a. The optimized transition state structures of COTh in S_0, S_1 and T_1. **b.** The chemical structure of COTh with bonds in the central ring labeled. **c.** Bond lengths of the central ring of COTh in S_0, S_1 and T_1.

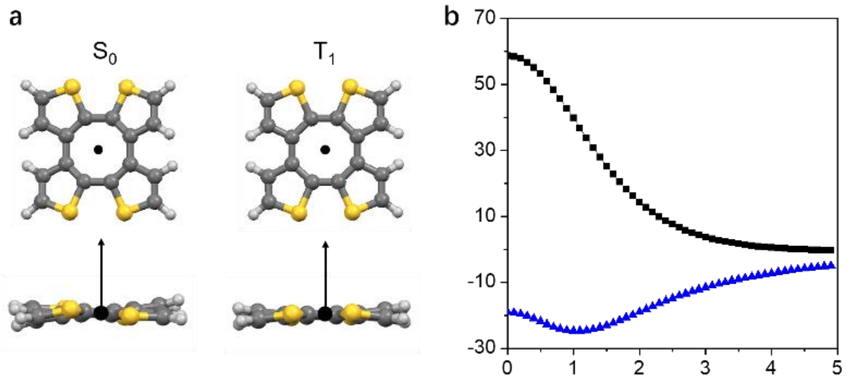

Supplementary Figure 32. Nucleus-independent chemical shift (NICS) scans of COTh of the transition state structures in the S0 and T1 states. a. $NICS_{zz}$ scans starting from the center of the central ring and scanned along the arrow. **b**. The curves recorded by $NICS_{zz}$ scan, black- and green-colored curves represent the $NICS_{zz}$ scans of COTh based on the S_0 and T_1 transition-state structures in the S_0 and T_1 states, respectively.

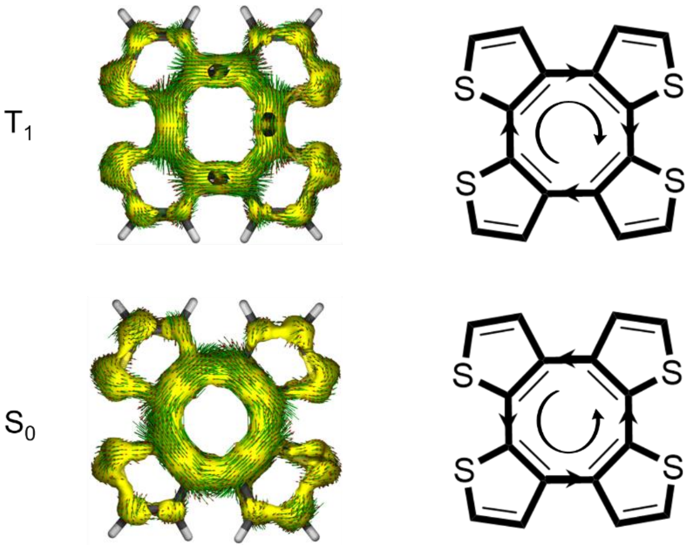

Supplementary Figure 33. Anisotropy of the induced current density (ACID) isosurfaces of the transition state structures in the S_0 and T_1 state. Arrows on the right side show the direction of the ring current, counterclockwise means antiaromaticity and clockwise means aromaticity.

Supplementary Table 7. The criteria to judge the aromaticity, anti-aromaticity and non-aromaticity (IUPAC).

	Aromatic	Anti-aromatic	Non-aromatic
criteria	cyclic<br>planar<br>conjugated<br>(4n+2) π electron	cyclic<br>planar<br>conjugated<br>(4n) π electron	fails any one of the 4 criteria

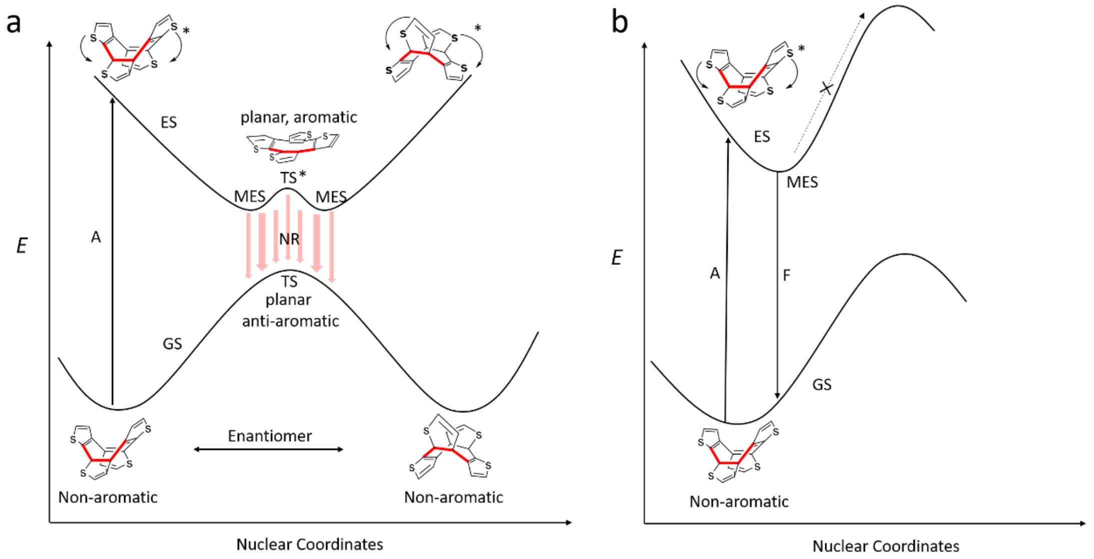

Supplementary Figure 34. a. Proposed decay pathways along the potential energy surface of COTh in dilute solution. **b**. Proposed decay pathways along the potential energy surface of COTh in solid state. Abbreviation: GS = ground state; ES = excited state; TS = transition state; MES = minimum energy structure; A = absorption; F = fluorescence; NR = Non-radiative decay.

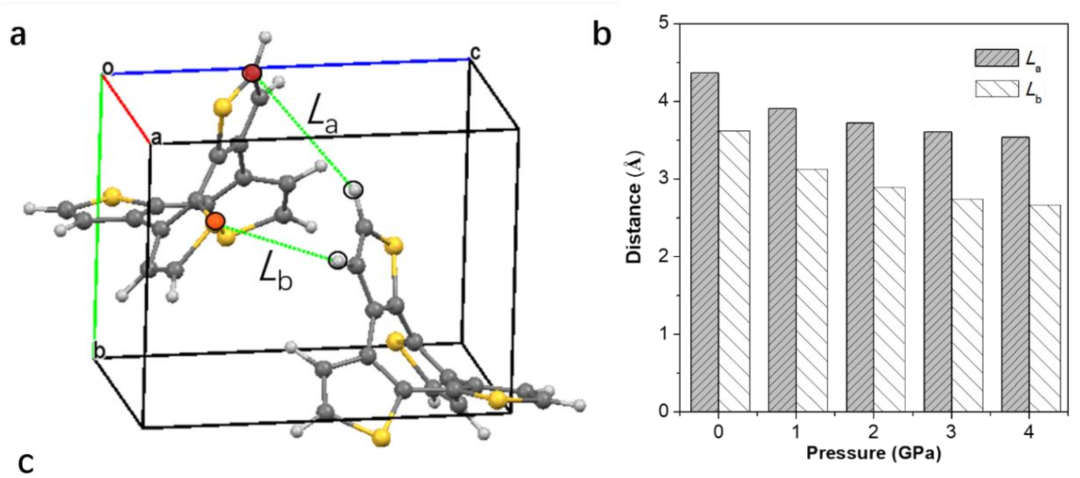

c

Pressure (GPa)	L_a	L_b
0	4.364	3.623
1	3.903	3.132
2	3.723	2.888
3	3.606	2.744
4	3.535	2.671

Supplementary Figure 35. Intermolecular distance of the crystal under varied pressure simulated by Material Studio. **a**, The investigated intermolecular distance (L_a and L_b) within the crystal of COTh. **b**, The change of L_a and L_b with hydrostatic pressure. **c**, Data summarization of L_a and L_b with hydrostatic pressure.

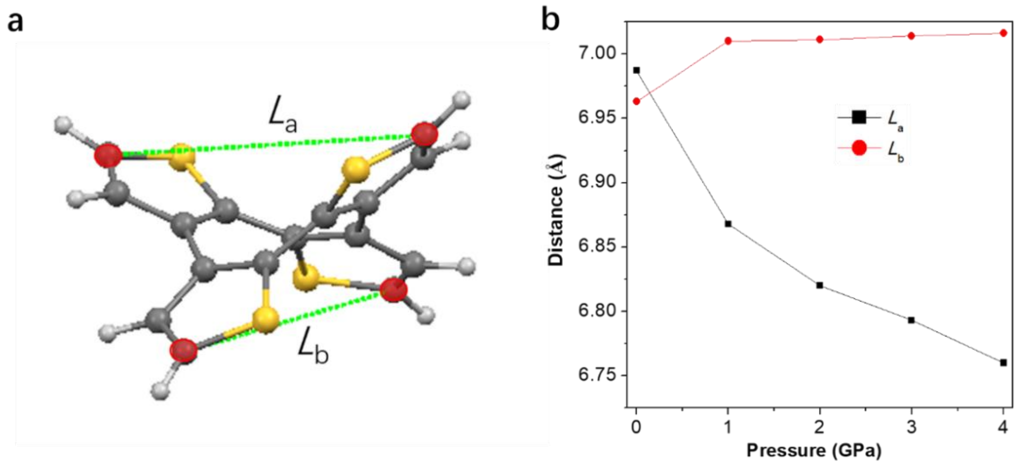

Pressure (GPa)	L_a	L_b
0	6.987	6.963
1	6.868	7.010
2	6.820	7.011
3	6.793	7.014
4	6.760	7.016

Supplementary Figure 36. Intermolecular distance of the crystal under varied pressure simulated by Material Studio. **a**, The investigated intramolecular distance (L_a and L_b) within the crystal of COTh. **b**, The change of L_a and L_b with hydrostatic pressure. **c**, Data summarization of L_a and L_b with hydrostatic pressure.

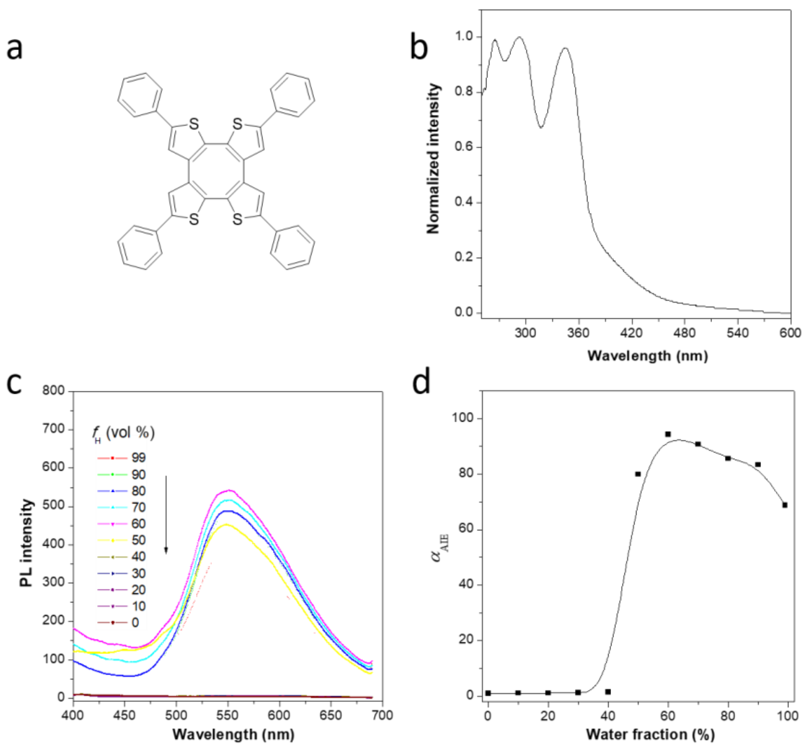

Supplementary Figure 37. Optical properties of COTh-Ph. a, Molecular structure of COTh-Ph. **b**, The absorption spectra of COTh-Ph in THF solution (c =10 μM) and thin film. **c**, PL spectra of COTh-Ph in THF/H_2O mixtures with different water fractions (f_w). **d**, The plot of the relative emission intensity ($\alpha_{AIE} = I/I_0$) versus the composition of the aqueous mixture of COTh-Ph. I_0 = PL intensity in pure THF.

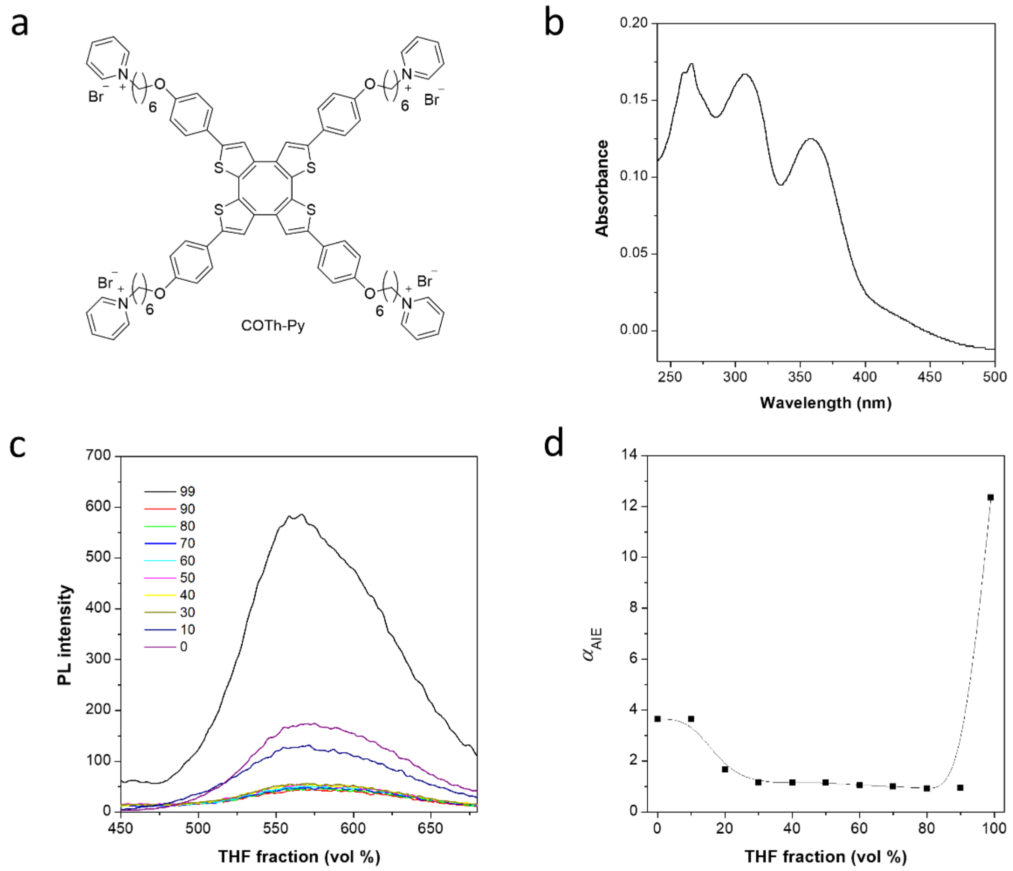

Supplementary Figure 38. Optical properties of COTh-Py. a, Molecular structure of COTh-Py. **b**, The absorption spectra of COTh-Py in aqueous solution (c =10 μM) and thin film. **c**, PL spectra of COTh-Ph in H_2O/THF mixtures with different THF fractions (f_T). **d**, The plot of the relative emission intensity (α_{AIE} = I/I_0) versus the composition of the aqueous mixture of COTh-Ph. I_0 = PL intensity in pure water.

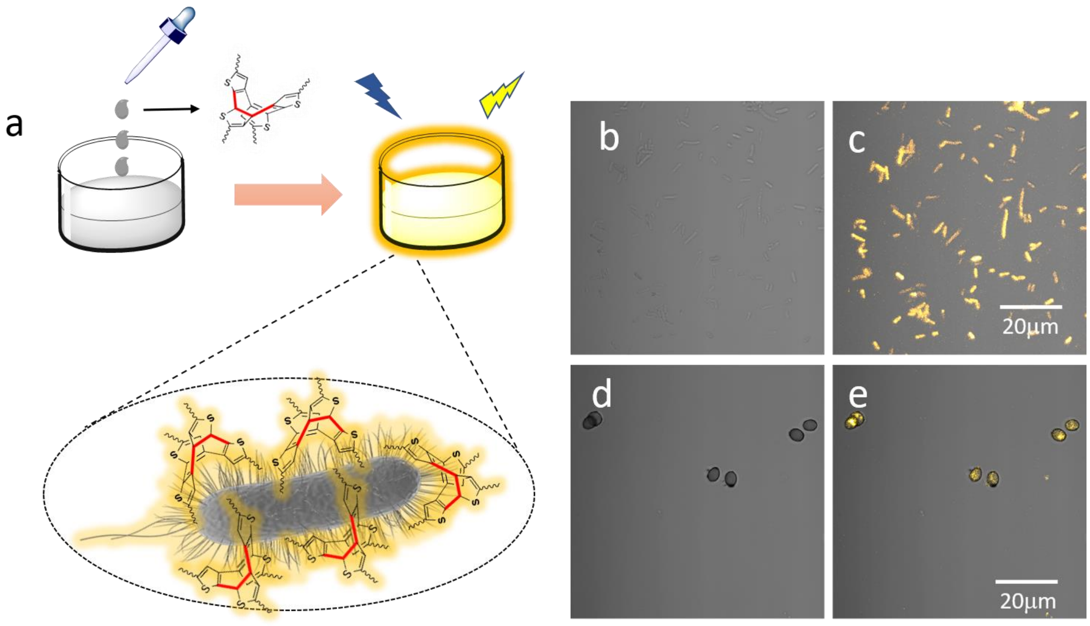

Supplementary Figure 39. **Microbia imaging application. a**, The cartoon illustrates the process of bacteria imaging. **b–c**, Bright-field and fluorescent images of *E. coli.* with 50μM of COTh-Py for 1 h. **d–e**, Bright-field and fluorescent images of P. chrysogenum with 20μM of COTh-Py for 2 h.

References

1. Wang, Y. *et al.* Derivation of saddle shaped cyclooctatetrathiophene: increasing conjugation and fabricating pentamer. *Tetrahedron* **70**, 631-636 (2014).
2. Wang, Y. *et al.* Efficient Synthesis of Trimethylsilyl-Substituted Dithieno[2,3-b:3′,2′-d]thiophene, Tetra[2,3-thienylene] and Hexa[2,3-thienylene] from Substituted [3,3′]Bithiophenyl. *Synlett,* **15**, 2390-2394 (2007).
3. Furche, F.; Ahlrichs, R.; Hättig, C.; Klopper, W.; Sierka, M.; Weigend, F., Turbomole. *Wiley Interdisciplinary Reviews: Computational Molecular Science* ***4***, 91-100 (2014).
4. Wang, J.; Wolf, R. M.; Caldwell, J. W.; Kollman, P. A.; Case, D. A., Development and testing of a general amber force field. *J. Comput. Chem.* **25**, 1157-1174 (2004).
5. Metz, S.; Kästner, J.; Sokol, A. A.; Keal, T. W.; Sherwood, P., ChemShell—a modular software package for QM/MM simulations. *WIRES Comput. Mol. Sci.***,** **4**, 101-110 (2014).

6. Smith, W.; Forester, T. R., DL_POLY_2.0: A general-purpose parallel molecular dynamics simulation package. *Journal of Molecular Graphics* **14**, 136-141 (1996).

7. Lin, H.; Truhlar, D. G., QM/MM: what have we learned, where are we, and where do we go from here? *Theor Chem Acc* **117**, 185-199 (2007).

8. Aaqvist, J.; Warshel, A., Simulation of enzyme reactions using valence bond force fields and other hybrid quantum/classical approaches. *Chem. Rev.* **93**, 2523-2544 (1993).

9. Lin, S. H.; Chang, C. H.; Liang, K. K.; Chang, R.; Shiu, Y. J.; Zhang, J. M.; Yang, T. S.; Hayashi, M.; Hsu, F. C., Ultrafast Dynamics and Spectroscopy of Bacterial Photosynthetic Reaction Centers. *Adv. Chem. Phys. 121*, 1-88 (2002).

10. Niu, Y.; Peng, Q.; Shuai, Z., Promoting-mode free formalism for excited state radiationless decay process with Duschinsky rotation effect. *Sci. China Ser. B Chem.* **51**, 1153-1158 (2008).

11. Peng, Q.; Yi, Y.; Shuai, Z.; Shao, J., Excited state radiationless decay process with Duschinsky rotation effect: Formalism and implementation. *J. Chem. Phys.* **126**, 114302 (2007).

12. Shuai, Z.; Peng, Q., Excited states structure and processes: Understanding organic light-emitting diodes at the molecular level. *Phys. Rep.* **537**, 123-156 (2014).

13. Peng, Q.; Niu, Y.; Shi, Q.; Gao, X.; Shuai, Z., Correlation Function Formalism for Triplet Excited State Decay: Combined Spin–Orbit and Nonadiabatic Couplings. *J. Chem. Theory Comput.***, 9**, 1132-1143 (2013).

14. Niu, Y.; Peng, Q.; Deng, C.; Gao, X.; Shuai, Z., Theory of Excited State Decays and Optical Spectra: Application to Polyatomic Molecules. *J. Phys. Chem. A***, 114**, 7817-7831 (2010).

15. Shuai, Z.; Peng, Q.; Niu, Y.; Geng, H., MOMAP, a free and open-source molecular materials property prediction package. *Revision 0.2.004; available online: http://www.shuaigroup.net/, Beijing,China.* **2014**.

16. Send, R.; Furche, F., First-order nonadiabatic couplings from time-dependent hybrid density functional response theory: Consistent formalism, implementation, and performance. *J. Chem. Phys.* **132**, 044107 (2010).

17. Geuenich, D.; Hess, K.; Köhler, F.; Herges, R. Anisotropy of the Induced Current Density (ACID), a General Method To Quantify and Visualize Electronic Delocalization. *Chem. Rev.*, **105**, 3758 (2005).
